# FMN reduces Amyloid-β toxicity in yeast by regulating redox status and cellular metabolism

**DOI:** 10.1038/s41467-020-14525-4

**Published:** 2020-02-13

**Authors:** Xin Chen, Boyang Ji, Xinxin Hao, Xiaowei Li, Frederik Eisele, Thomas Nyström, Dina Petranovic

**Affiliations:** 1 Division of Systems and Synthetic Biology, Department of Biology and Biological Engineering, Chalmers University of Technology, SE41296 Gothenburg, Sweden; 2 Novo Nordisk Foundation Center for Biosustainability, Chalmers University of Technology, SE41296 Gothenburg, Sweden; 3 Institute for Biomedicine, Sahlgrenska Academy, Centre for Ageing and Health-AgeCap, University of Gothenburg, SE40530 Gothenburg, Sweden

**Keywords:** Protein folding, Microbiology, Systems biology

## Abstract

Alzheimer’s disease (AD) is defined by progressive neurodegeneration, with oligomerization and aggregation of amyloid-β peptides (Aβ) playing a pivotal role in its pathogenesis. In recent years, the yeast *Saccharomyces cerevisiae* has been successfully used to clarify the roles of different human proteins involved in neurodegeneration. Here, we report a genome-wide synthetic genetic interaction array to identify toxicity modifiers of Aβ42, using yeast as the model organism. We find that *FMN1*, the gene encoding riboflavin kinase, and its metabolic product flavin mononucleotide (FMN) reduce Aβ42 toxicity. Classic experimental analyses combined with RNAseq show the effects of FMN supplementation to include reducing misfolded protein load, altering cellular metabolism, increasing NADH/(NADH + NAD^+^) and NADPH/(NADPH + NADP^+^) ratios and increasing resistance to oxidative stress. Additionally, FMN supplementation modifies Htt103QP toxicity and α-synuclein toxicity in the humanized yeast. Our findings offer insights for reducing cytotoxicity of Aβ42, and potentially other misfolded proteins, via FMN-dependent cellular pathways.

## Abstract (web summary)

*Saccharomyces cerevisiae* is a model organism to study proteins involved in neurodegeneration. Here, the authors performed a yeast genome-wide synthetic genetic interaction array (SGA) to screen for toxicity modifiers of Aβ42 and identify riboflavin kinase and its metabolic product flavin mononucleotide as modulators that alleviate cellular Aβ42 toxicity, which is supported by further experimental analyses.

## Introduction

Alzheimer’s disease (AD) is the most common neurodegenerative disorder in aging populations. It is categorized as a protein misfolding, or protein conformational disease due to the accumulation of misfolded amyloid-β (Aβ) peptides, which are defined as one of its principal hallmarks^1^. The aggregation of Aβ is thought to be an early driver of AD, triggering a cascade of pathogenic processes, such as inflammation, neurofibrillary tau-tangle formation, and synapse dysfunction^2^. Brain extracts from AD patients or transgenic mice containing Aβ have also shown to accelerate cerebral Aβ plaque accumulation and associated pathology in genetically modified mice^3^. Aβ42, which is two amino acids longer than the usual form of the peptide (Aβ40), is more hydrophobic and prone to form aggregates, being enriched in patients diagnosed with AD^4^. Nonetheless, despite our understanding of AD pathogenesis tremendously advancing over the past three decades, efforts to translate this knowledge into effective therapies have, so far, had limited success^5^.

As a principal organelle responsible for protein quality control and secretion, the endoplasmic reticulum (ER) is dramatically perturbed in AD neurons^6^. Increased Aβ production and/or aggregation is proposed to result in abnormal levels of ER stress, contributing towards synapse dysfunction in AD^7^. To restore cellular proteome homeostasis (proteostasis), ER stress can activate the unfolded protein response (UPR)^8^, which in turn promotes the proper folding of misfolded proteins and suppresses translation, preventing an overload of the ER with newly synthesized proteins. As well, UPR leads to an activation of protein degradation via the ER-associated protein degradation (ERAD) pathway^9,10^. However, under chronic or irreversible ER stress conditions, the UPR shifts its signaling toward cell death^11^. Modulation of ER-UPR has therefore been one potential therapeutic strategy for slowing down AD, using gene therapy or pharmacological approaches^12,13^.

Due to the strong conservation of protein quality control systems among eukaryotic cells, the yeast *Saccharomyces cerevisiae* has become a powerful model organism to study misfolded proteins and their implication in human pathologies. Many available molecular and genetic tools, alongside defined media, and ease of handling provide many benefits for using this model system for high-throughput genetic and drug candidate screenings^14,15^. Indeed, several humanized yeast models have been successfully developed to study different aspects of Aβ toxicity. In humans, Aβ peptides are generated through the sequential cleavage of the amyloid precursor protein (APP)^16^ by BACE and γ-secretase^17^, which occurs in the secretory pathway and endocytic pathway in neurons^18^. In yeast, these human Aβ peptides can be co-expressed with an ER Kar2 signal peptide, to ensure that Aβ peptides are transited through the secretory pathway and exported from the cytoplasm. However, as the yeast cell wall prevents secreted Aβ from diffusing away extracellularly, these Aβ peptides re-enter into the cell through endocytosis. Aβ42 peptides subsequently form more oligomers relative to Aβ40 and exhibit an increased cellular toxicity in yeast^19,20^.

To mimic chronic cytotoxicity during AD progression, we recently developed constitutive Aβ42 and Aβ40 expression models in yeast^21^. The constitutive Aβ42 expression provides a model for moderate and cumulative effects of Aβ42 toxicity, over a longer period, in conditions of aging yeast cells. Similar to previous observations in human neurons and other AD model organisms^22^, we found that constitutive expression of Aβ42 peptides forms more toxic oligomers and triggers a stronger ER stress response and UPR, compared to the expression of less toxic Aβ40 peptides^23^. We also observed reduced mitochondrial function and an increase in the production of reactive oxygen species (ROS), alongside a redirection of energy from cell growth to maintenance^23^.

The synthetic genetic array (SGA) approach has been applied to yeast neurodegeneration models, such as AD, Huntington’s disease (HD)^24^ and Parkinson’s disease (PD)^25^, demonstrating its robust capability to help identify genes and mechanisms underlying phenotypes of interest, such as modeling toxicity of human disease proteins. In previous studies, the nonessential genome-wide deletion library has been applied to Aβ42 expression strain to identify proteins and cellular processes affecting intracellular Aβ42 aggregation and toxicity^26,27^. The screening of GFP fused Aβ42 (Aβ42-GFP) identified four major cellular processes as being significantly overrepresented: mitochondrial function, phospholipid metabolism, transcriptional/translational regulation and inositol biosynthesis^26^. Another screening of Aβ42 strain with a α-prepro factor sequence revealed that ER–Golgi traffic, mitochondrial functioning, cell cycle, DNA replication stress response and ESCRT machinery play vital roles in Aβ42 toxicity^27^. In both studies, expression of Aβ42 peptides is under the control of a strong inducible promoter, whereas the Aβ42 production in neurons is constitutive. The inducible promoter allows for well-timed induction of acute cytotoxicity but is accompanied by a drastic change in carbon-source and metabolism.

Here we take advantage of our improved Aβ42 humanized yeast model to investigate genetic interactions that can provide insights into the Aβ42 cytotoxic phenotype. We applied SGA technology to screen for genetic mutants with an altered Aβ42 sensitivity profile, by using a yeast deletion mutant library in which we constitutively expressed Aβ42. The two mutant libraries comprise a collection of ~4300 deletion strains for nonessential genes (SGA-V2) and ~1200 temperature sensitive mutant strains for essential genes (ts-V6). These two sets together represent more than 80% of all yeast genes^28^. Compared with previous studies, inclusion of essential genes screening in our SGA analysis may further exploit the cellular mechanisms affecting Aβ42 toxicity. This unbiased yeast genome-scale screen thus enables a global analysis of synthetic genetic interactions, which enabled us to identify mechanistic connections between genes and their corresponding pathways^29^. Using the tool in this study, it was therefore possible to map the genome-wide interaction networks involved in Aβ42 toxicity. From the essential mutant library, we identified the riboflavin kinase gene (*FMN1*) and its metabolic product flavin mononucleotide (FMN) as being able to alleviate cellular Aβ42 toxicity.

## Results

### Yeast genome-wide screening for modulators of Aβ42 toxicity

**Fig. 1:**
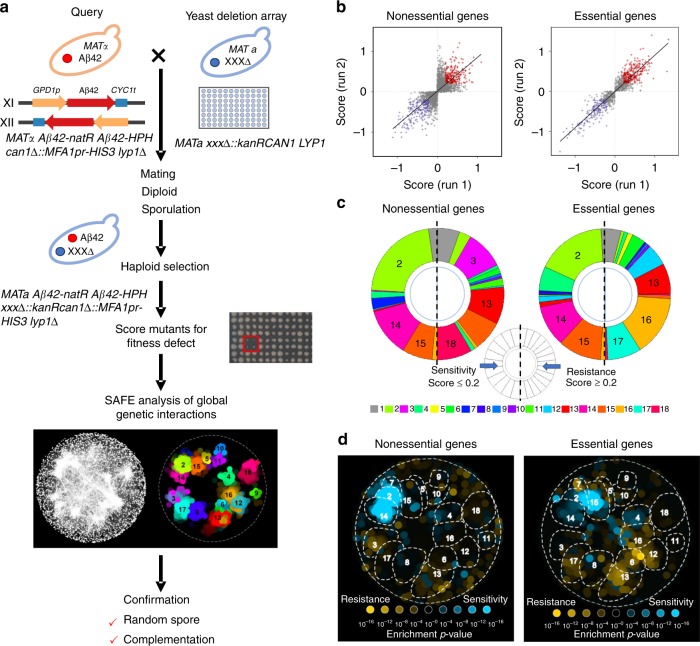
Genome-wide synthetic genetic array (SGA) screen to identify modulators of Aβ42 toxicity. **a** A schematic workflow for identifying mutants that can alter Aβ42 toxicity. **b** Scores for each genetic mutant, from two independent experiments, with those that significantly change Aβ42 toxicity indicated in red (*p*-adj < 0.05, score ≥ 0.2), and blue (*p*-adj < 0.05, score ≤ −0.2), respectively. **c** Gene ontology (GO) biological process term distributions among mutant strains with significantly affected Aβ42 toxicity (*p*-adj < 0.05, score ≥ 0.2 or ≤−0.2). Each number corresponds to a different biological process GO term as follows: 1. Other; 2. Glycosylation, protein folding/targeting, cell wall biosynthesis; 3. Ribosome biogenesis; 4. Protein degradation; 5. Cytokinesis; 6. Nuclear-cytoplasmic transport; 7. Multivesicular body (MVB) sorting and pH-dependent signaling; 8. mRNA processing; 9. tRNA wobble modification; 10. Peroxisome; 11. Metabolism and fatty acid biosynthesis; 12. DNA replication and repair; 13. Transcription and chromatin organization; 14. Vesicle traffic; 15. Cell polarity and morphogenesis; 16. Mitosis and chromosome segregation; 17. rRNA and ncRNA processing; 18. Respiration, oxidative phosphorylation, mitochondrial targeting. The top 3 GO terms are indicated as number in each individual category. **d** Spatial analysis of functional enrichment of mutants with significantly (*p*-adj < 0.05) affected Aβ42 toxicity and score ≥ 0.2 or ≤−0.2. Blue colored points indicate mutants more sensitive to Aβ42 toxicity (score ≤ −0.2) and yellow color indicates mutants that are more resistant (score ≥ 0.2), as measured via their growth during SGA screening. GO biological process terms are indicated as in **c** and outlined by dashed lines.

Our humanized Aβ42 yeast model was constructed with a centromeric plasmid under the control of a constitutive *GPD* promoter, with the Kar2 signal sequence in front of the Aβ42 sequence^21^. To minimize the experimental variation during high-throughput screening, we integrated two copies of the Kar2-Aβ42-encoding sequence under the control of the *GPD* promoter into the SGA starting strain (Y7092) (Supplementary Fig. 1), and tested it to confirm that the previously characterized phenotypes were preserved. The main reason for using the Y7092 strain being that it incorporates reporters and markers necessary for SGA haploid strain selection, following meiotic recombination. To create an SGA control query strain, only the promoter and terminator sequences were integrated into the Y7092 strain (Supplementary Fig. 1). The query strains were mated with the yeast deletion mutant library to generate two arrays, where each deletion strain on the array was combined with either the control or Aβ42 expression. The scores for growth defects were generated through comparing the colony sizes of mutants with the Aβ42 expression to mutants with the control (Fig. 1a). Scores < 0 or >0 represent either a decrease or an increase of growth in mutants with Aβ42 expression.

The SGA screen was performed independently twice. The cutoffs for scores were set at ≤−0.2 or ≥ 0.2 (*p*-adj < 0.05, Supplementary Fig. 2 and Supplementary Fig. 3). Among the analyzed 4,300 mutants for nonessential genes, 229 were scored ≤ −0.2, that is these mutants showed higher sensitivity to Aβ42 toxicity and 259 were scored ≥ 0.2, that is these mutants showed higher resistance to Aβ42 toxicity, in both SGA tests as seen through their difference in growth profiles (Fig. 1b and Supplementary Data 1). The analysis of the 1,200 mutants for essential genes revealed 198 to have a score ≤ −0.2 and 253 had a score ≥ 0.2 (Fig. 1b and Supplementary Data 2). The gene set enrichment analysis (GSEA) showed that sensitive mutants (decreased growth with Aβ42 expression, score ≤ −0.2) significantly enriched in the vesicle-mediated transport (*p* = 7.94e-10), glycosylation (*p* = 8.01e-5) and response to unfolded protein (*p* = 9.63e-6) in nonessential collection (Supplementary Fig. 4). While in essential collection, the sensitive mutants significantly enriched in the vesicle-mediated transport (*p* = 1.55e-8), protein N-linked glycosylation (*p* = 4.58e-6) and autophagy (*p* = 2.52e-4) (Supplementary Fig. 5). Furthermore, we performed a functional enrichment analysis via spatial analysis of functional enrichment (SAFE)^30^. The enrichment of GO terms suggested that 44% and 34% of the genes mutated in the sensitive mutants in nonessential and essential collections, respectively, belonged to group 2 (glycosylation, protein folding/targeting, cell wall biosynthesis processes) (enrichment *p* value < 0.05). Similarly, 24% and 21% of the genes mutated in the sensitive mutants in nonessential and essential collections, respectively, were represented by group 14 (vesicle traffic processes) (enrichment *p* value < 0.05, Fig. 1c, d). Moreover, the enrichment of GO terms for resistant mutants that had increased growth with Aβ42 expression (score ≥ 0.2) was represented in different biological processes in both mutant collections (Fig. 1c, d). Besides these results, among the essential genes required for Aβ42 tolerance, many belonged to group 4 (protein degradation processes) as well as group 15 (cell polarity and morphogenesis processes). Essential genes, which mutations caused increased resistance against Aβ42, were enriched in group 13 (transcription and chromatin organization processes), group 16 (mitosis and chromosome segregation processes) and group 17 (rRNA and ncRNA processing processes) (enrichment *p* value < 0.05, Fig. 1c, d). The complete list of GO terms from SAFE analysis can be found in Supplementary Tables 1 and 2.

### Yeast mutants with increased sensitivity to Aβ42 toxicity

**Fig. 2:**
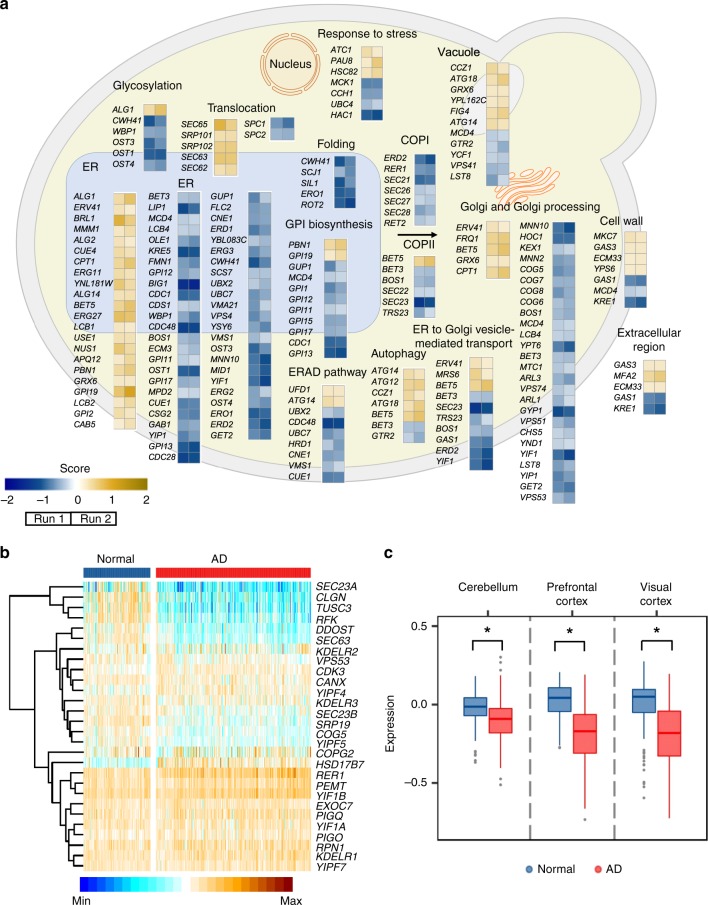
Roles of protein secretion and degradation processes in Aβ42 toxicity. **a** Mutants with significantly changed Aβ42 toxicity mapped to protein secretory and degradation processes (*p*-adj < 0.05 and score ≥ 0.2 or ≤−0.2). **b** The mRNA expression levels of 28 human orthologs in the prefrontal cortex of Alzheimer’s disease (AD) patients versus normal controls. Genes were ranked according to the size of their significant difference between the two groups. Out of the 28 human orthologs, 22 (79%) showed significantly changed mRNA levels (*p*-adj < 0.05). **c** Comparison of human *RFK* (ortholog of yeast *FMN1*) mRNA levels in three different brain regions (cerebellum, prefrontal cortex and visual cortex) between AD patients and normal controls. Asterisks (*) indicate significant differences (*p*-adj < 0.0001).

Gene set enrichment analysis revealed that Aβ42 toxicity was found to be increased in mutants involved in protein secretion and degradation processes, including protein translocation, posttranslational modifications, folding to a mature protein, ER to Golgi transport, ER to vacuole transport, ERAD and autophagy. To visualize the functions of these mutated genes, we mapped all mutants that met our significance and growth score cut offs (*p*-adj < 0.05, score ≤ −0.2 or ≥0.2) on a schematic representation of protein secretion and degradation pathways map (Fig. 2a). These findings are in accordance with our previous findings from genome-wide transcriptional analysis, which showed that Aβ42 expression triggers a strong ER stress, resulting in activation of the UPR to upregulate processes involved in protein folding/maturation, ER-to-Golgi trafficking and ERAD^23^ (Supplementary Fig. 6).

By selecting more stringent cutoff values (*p*-adj < 0.05, score ≤ −0.5 or ≥0.5), 47 sensitive mutants and 7 resistant mutants were identified (listed in Supplementary Tables 3 and 4, respectively). Interestingly, 35 out of these 54 mutants were determined in human (HomoloGene, https://www.ncbi.nlm.nih.gov/homologene). To assess the potential clinical relevance of these genes, we further examined the mRNA expression of the corresponding human orthologs in the prefrontal cortex of AD patients versus controls (healthy) samples, which enabled us to examine 28/35 human ortholog genes. We discovered that 14 and 8 out of the 28 human orthologs showed significantly decreased and increased mRNA levels, respectively (Fig. 2b and Supplementary Fig. 7). We further manually tested the phenotypes of the 28 corresponding yeast mutants, of which 13 mutant phenotypes were confirmed by random spore test (Table 1).

**Table 1:** Modifiers of Aβ42 toxicity in protein secretion and degradation processes^a^.

Systematic name	Standard name	Cellular function	Human ortholog
*YAL058W*	*CNE1*	Folding and quality control of glycoproteins	*CANX*/*CLGN*
*YOR085W*	*OST3*	Oligosaccharyltransferase	*TUSC3*
*YDR236C*	*FMN1*	FMN biosynthesis	*RFK*
*YEL002C*	*WBP1*	N-linked glycosylation of proteins in the ER	*DDOST*
*YOR254C*	*SEC63*	Target and import proteins into ER	*SEC63*
*YBL040C*	*ERD2*	Maintenance of normal levels of ER-resident proteins	*KDELR1*/*KDELR2*/*KEDLR3*
*YGR172C*	*YIP1*	Biogenesis of ER-derived COPII transport vesicles	*YIPF4*/*YIPF5*/*YIPF7*
*YNL287W*	*SEC21*	ER to Golgi transport of selective cargo	*COPG2*
*YLR100W*	*ERG27*	Ergosterol biosynthesis	*HSD17B7*
*YCL001W*	*RER1*	Retention of membrane proteins to ER	*RER1*
*YGR216C*	*GPI1*	Synthesis of GlcNAc-PI	*PIGQ*
*YLL031C*	*GPI13*	ER membrane localized phosphoryltransferase	*PIGO*
*YJL002C*	*OST1*	Oligosaccharyltransferase	*RPN1*

^a^Yeast genes with human orthologs were identified in SGA screening and confirmed by random spore test.

Six out of these 13 mutants had functions related to ER and glycosylphosphatidylinositol (GPI) biosynthesis (*FMN1*, *YIP1*, *CNE1*, *ERG27, GPI13*, and *GPI1*), four were related to glycosylation and translocation (*WBP1*, *OST1*, *OST3*, *SEC63*), and three were functionally associated with COPI transport (*SEC21*, *ERD2*, *RER1*). Interestingly, of these human orthologs, four (*SEC63*^31^, *KDELR1*^32^, *RER1*^33^, and *RPN1*^34^), which are the orthologs of yeast *SEC63*, *ERD2*, *RER1*, and *OST1*, respectively, have already been identified in previous studies for association with AD^31–34^.

### Defects in riboflavin metabolism increases Aβ42 toxicity

Among these confirmed mutants with human orthologs, we found the *FMN1* gene. *FMN1* encodes an essential enzyme that is responsible for catalyzing the phosphorylation of riboflavin (vitamin B_2_), in turn to produce the active forms of this vitamin^35^. Riboflavin has been found to act as a potential neuroprotective agent, preventing and/or modifying the process of neurological disorders by improving conditions of oxidative stress, mitochondrial dysfunction, and neuroinflammation^36^. Moreover, mRNA levels of human *RFK* (riboflavin kinase), the ortholog of yeast *FMN1*, have found to be significantly lower in the cerebellum, visual cortex and prefrontal cortex of AD patients versus controls (Fig. 2c).

**Fig. 3:**
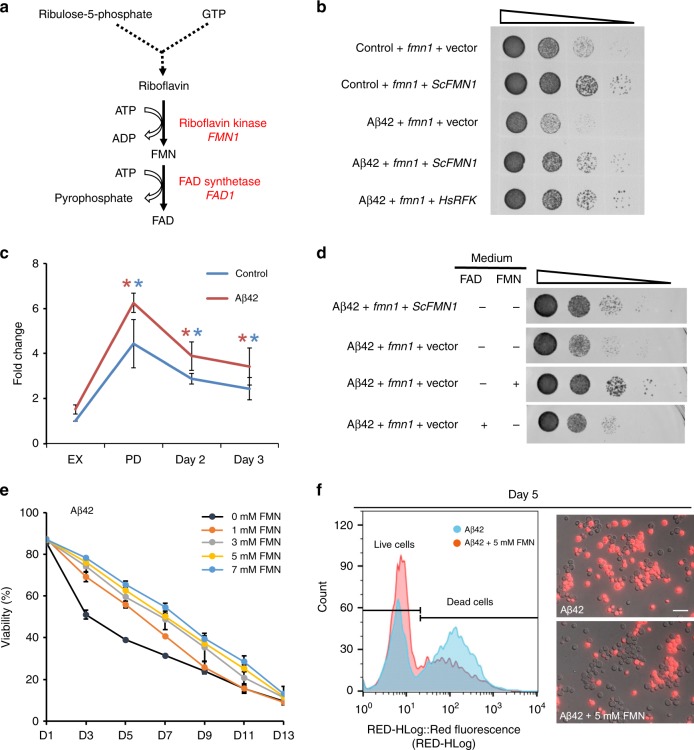
Riboflavin kinase and its metabolic product FMN reduce Aβ42 toxicity. **a** Schematic illustration of the riboflavin biosynthesis metabolic pathway. FMN, flavin mononucleotide; FAD, flavin adenine dinucleotide. **b** Complementation assays to confirm *fmn1* mutant is causal towards increasing Aβ42 sensitivity. Vector: MoBY empty plasmid; *ScFMN1*: pFMN1-MoBY plasmid; *HsRFK*: P416 GPD-human RFK plasmid. **c** *FMN1* mRNA expression during exponential growth (EX), post-diauxic shift phase (PD) and stationary phase (day 2 and day 3). Results from qPCR are normalized to EX of each strain and shown as the average values ± SD from three independent biological replicates. Asterisks (*) indicate significant differences compared to EX in both Aβ42 (red) and control (blue) strains (*p* < 0.05). **d** Ten-fold serial dilutions of the Aβ42 expression strain with *fmn1* mutation carrying the aforementioned plasmids with supplementation of 5 mM FAD or 5 mM FMN in the medium. **e** Survival of the Aβ42 strain during stationary phase with different concentrations of FMN supplemented. Viability is shown as the fraction of propidium iodide (PI) negative cells (*n* = 3 ± SD). **f** Flow cytometry and fluorescence micrographs of PI staining in the Aβ42 strain after 5 days of cultivation. Cells that are stained red represent nonviable cells. Scale = 10 µm. Source data are provided as a Source Data file.

Riboflavin biosynthesis in *S. cerevisiae* utilizes ribulose 5-phosphate and guanosine-5′-triphosphate (GTP) as precursors and proceeds through pyrimidine intermediates^37^. The active forms of riboflavin, FMN and flavin adenine dinucleotide (FAD), are formed in two consecutive reactions which are catalyzed by FMN1 and FAD1 (FAD synthetase), respectively (Fig. 3a). The SGA screening showed that the *fmn1* mutant was sensitive to Aβ42 toxicity with a score of −0.55 and −0.66 from the two independent SGA screens. Moreover, complementation assays confirmed that *fmn1* mutant was crucial for Aβ42 toxicity. As well, increased Aβ42 toxicity in the *fmn1* mutant background could be reduced by expression of *FMN1* or the human ortholog *RFK* (Fig. 3b and Supplementary Fig. 8). We further measured the dynamic transcript levels of *FMN1* in both Aβ42 and control strains. The results showed that *FMN1* mRNA levels were significantly increased in the post-diauxic shift (PD) and stationary phases (SP) cells compared to exponentially growing cells (EX) in both Aβ42 and control strains (*p*-adj < 0.05, Fig. 3c). In the Aβ42 strain, there was 4.10, 2.55, 2.25-fold increase in PD, day 2, and day 3 compared to EX, respectively. In the control strain, there was 4.4, 2.9, and 2.4-fold increase in PD, day 2, and day 3 compared to EX, respectively (Fig. 3c).

### FMN supplementation reduces Aβ42 toxicity

To test whether the increased Aβ42 toxicity was due to the *fmn1* mutation or to a lack of downstream metabolic products, we cultivated the Aβ42 strain with *fmn1* mutation in culture medium containing either 5 mM FMN or 5 mM FAD, a metabolic product that is one-step down from FMN in the riboflavin biosynthetic pathway. Here, the Aβ42 toxicity was only found to be reduced by supplementation with 5 mM FMN, not FAD (Fig. 3d). As well, supplementation with FMN could also rescue the growth impairment of the control strain with *fmn1* mutation (Supplementary Fig. 9). As a riboflavin-nucleotide coenzyme, FMN is essential for redox reactions and participates in a wide range of biological processes, including carbohydrate, fatty acid and amino acid metabolisms^38^. These observations suggest that maintaining a sufficient level of FMN may be required for both Aβ42 and control strains during different stages of cell growth. Furthermore, these results also suggest FMN deficiency could contribute towards Aβ42-triggered toxicity. To test this, we evaluated cellular viability in the presence of different concentrations of FMN in the culture medium following chronological aging. The significantly increased viability of Aβ42 strain was FMN-dosage dependent (Fig. 3e, f). The fractions of non-viable cells on day 5 were 61% in Aβ42 strain without FMN, comparing to 45%, 39.4%, 35%, and 33.3% with 1, 3, 5, and 7 mM FMN added, respectively (Supplementary Fig. 10). Moreover, supplementation of FMN also improved viability in the control strain as well (*p*-adj < 0.05, Supplementary Fig. 11).

### FMN supplementation plays important roles in protein folding

**Fig. 4:**
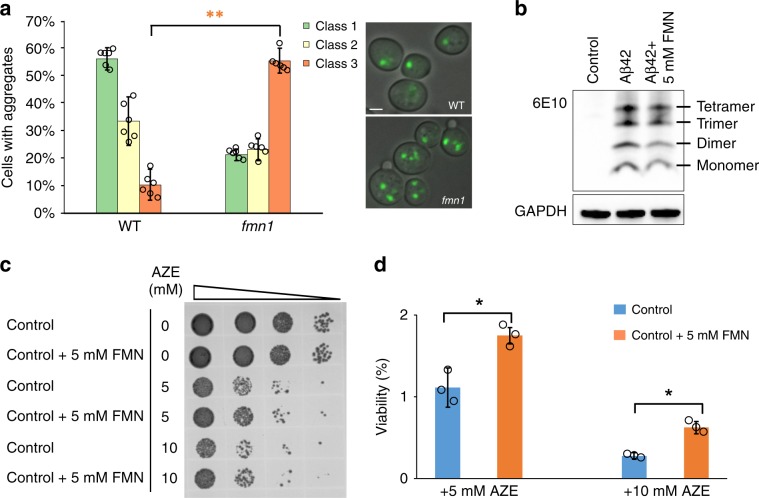
FMN supplementation facilitates protein folding. **a** Quantification of aggregate foci after heat shock for 90 min in wild type and *fmn1* mutant yeast cells. Class1: one aggregate per cell (as highlighted by single GFP cluster); Class 2: two aggregates per cell; Class 3: ≥3 aggregates per cell. Bar graphs show fractions of Class 1, 2, and 3 cells for each strain. Above 500 cells were measured per sample with results shown as average values ± SD from six biological experiments. Scale = 2 µm. **p < 0.001. **b** Western blot of Aβ42 expression from stationary phase cultures, using the 6E10 Aβ specific antibody. GAPDH was used as the loading control. **c** Ten-fold serial dilutions of the control strain after 3 h of treatment with 5 or 10 mM L-azetidine-2-carboxylic acid (AZE). **d** Colony forming unit (CFU) measurement of control strain after 5 mM or 10 mM of AZE treatment as a measurement of viability (*n* = 3 ± SD). *p < 0.05. Source data are provided as a Source Data file.

Increasing evidence indicates that Aβ42 peptides are prone to form aggregates, which contributes notably to the neurotoxicity that occurs in AD pathogenesis^39^. To monitor whether the *fmn1* mutation contributed to protein aggregation, we used GFP-tagged Hsp104 (heat shock protein 104) as a reporter for protein aggregation. In *S. cerevisiae*, Hsp104 plays a crucial role in thermotolerance, binding to aggregated proteins after exposure to heat shock^40^. The GFP-tagged Hsp104 can therefore be observed as intracellular foci and inclusions using fluorescence microscopy^41^. Cells from both the *fmn1* mutant and the wild type were subjected to heat shock at 38 °C for 90 min to induce protein misfolding and aggregation. Here, *fmn1* mutant was found to contain significantly (*p* < 0.001) more cells in the class 3 category (55.5 ± 4.6%, ≥3 aggregates/cell), than in the wild type strain (10.4 ± 5.7%, Fig. 4a). This indicates that *FMN1* could be involved in sequestering small aggregates/oligomers into larger inclusion bodies, as part of a protective response towards reducing proteotoxicity^42^. Correspondingly, we observed that 5 mM FMN supplementation decreased 21% of aggregate formation in Aβ42 strain, which links to Aβ42 induced toxicity (Fig. 4b).

To further investigate if FMN supplementation can help cells to cope with a protein misfolding burden, we again stressed the cells, but this time directly targeted proteins as opposed to using general stress conditions. To achieve this, we used l-azetidine-2-carboxylic acid (AZE), an analog of l-proline which causes protein misfolding. In the control strain, we observed that cellular viability was significantly (*p* < 0.05) decreased with 5 mM and 10 mM AZE treatment, but cells with 5 mM FMN supplementation showed improved survival than without supplementation (Fig. 4c). Here, the number of viable cells was measured by colony forming units (CFU), which showed significantly (*p* < 0.05) higher viability in FMN supplemented cultures with either 5 or 10 mM AZE treatment compared to cultures without FMN (Fig. 4d). This result indicates that FMN supplementation could increase cellular capacity to cope with misfolded protein stress.

### FMN improves redox homeostasis in Aβ42 strain

**Fig. 5:**
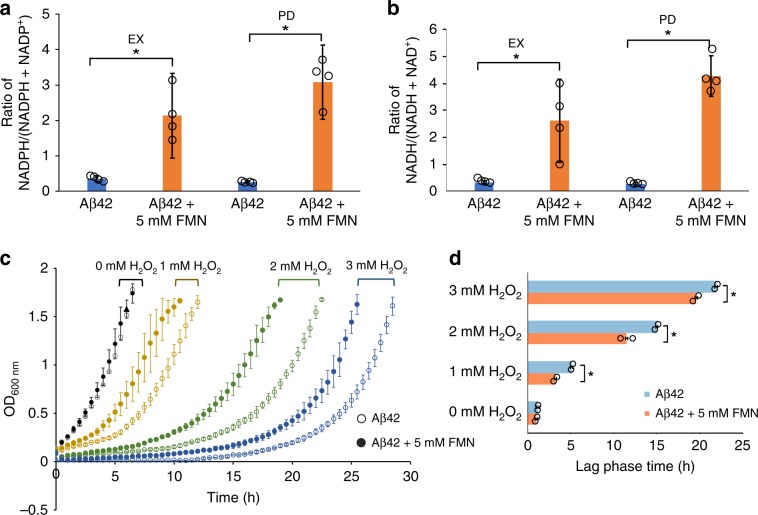
FMN supplementation increases cellular tolerance to oxidative stress in Aβ42 strain. **a** Ratio of NADPH/(NADPH + NADP^+^) in the Aβ42 strain without or with FMN supplementation during EX and PD growth phases respectively (*n* = 4 ± SD; *P < 0.01). **b** Ratio of NADH/(NADH + NAD^+^) in the Aβ42 strain without or with FMN supplementation during EX and PD growth phases (*n* = 4 ± SD; *P < 0.01). **c**, **d** Aβ42 cells display an increased H_2_O_2_ tolerance when supplemented with FMN. Aβ42 cells without or with FMN supplement were grown to mid exponential phase (OD ≈  0.5–0.6) and treated with different concentrations of H_2_O_2_. Cell growth was monitored using a microplate reader (**c**) with lag phase time analyzed using the R package growthrates (**d**). The lag phase indicates the time from H_2_O_2_ treatment until maximum growth rate, determined by the exponential growth phase. Results shown are average values ± SD of biological duplicates. The asterisk (*) indicates significant difference (*p* < 0.05). Source data are provided as a Source Data file.

Oxidative stress has been implicated in many disease processes including neurodegenerative disorders and aging^43^. A balanced ratio of cellular oxidants and antioxidants is important to maintain a state of redox homeostasis, which is mainly determined by the ratios between reduced and oxidized forms of redox cofactors, particularly nicotinamide adenine dinucleotide phosphate (NADP) and nicotinamide adenine dinucleotide (NAD). In the Aβ42 strain, 5 mM FMN supplementation resulted in a significant (*p* < 0.05) increase of the NADPH/(NADPH + NADP^+^) ratio during EX and PD conditions (Fig. 5a and Supplementary Fig. 12). Under these conditions, NADH/(NADH + NAD^+^) ratios were also significantly (*p* < 0.05) improved as well (Fig. 5b and Supplementary Fig. 12). Finally, FMN supplementation was also found to increase the NADPH/(NADPH + NADP^+^) ratio in the control strain without Aβ42 expression (*p* < 0.05, Supplementary Figs. 12 and 13a). As oxidative stress has been implicated as one of causative factors for aging^44^, the improved reducing environment might contribute to the increased viability in the control strain during chronological aging (Supplementary Fig. 11). Therefore, as well as lessening the propensity for protein misfolding, FMN supplementation may also alleviate Aβ42 toxicity through improving redox homeostasis which can help overall cell response to oxidative stress.

To investigate this, we challenged the Aβ42 strain with the endogenous oxidant hydrogen peroxide (H_2_O_2_). Here, cells were grown to mid exponential phase and exposed to incremental levels of H_2_O_2_. The length of the lag phase was calculated from exponential growth curves, by calculating the time after cells were arrested in growth following H_2_O_2_ treatment to the point where they returned to maximum growth rate^45^. We observed that Aβ42 cells displayed prolonged lag phases with higher H_2_O_2_ concentrations. However, the supplementation of 5 mM FMN significantly (*p* < 0.05) shortened the extended lag phases (Fig. 5c, d). An oxidative stress tolerance assay for the control strain was also performed using spot tests with medium containing different concentrations of H_2_O_2_. As seen with the liquid growth assay, cells with 5 mM FMN supplementation demonstrated a higher H_2_O_2_ tolerance than without supplementation (Supplementary Fig. 13b). Taken together, these results indicate that FMN supplementation can be beneficial for oxidative stress tolerance, in both Aβ42 and control strains.

### FMN supplementation causes a global transcriptional response

To gain further insight into the mechanism behind how FMN supplementation helps to improve cell physiology, Aβ42 and control strains with and without 5 mM FMN supplementation were sampled for RNA sequencing (Supplementary Fig. 12). Principal component analysis (PCA) showed that the Aβ42 strain with FMN supplementation had a distinct gene expression profile (Supplementary Fig. 14a). Differential gene expression analysis identified more than a third of the genome (*n* = 2081 genes) to be significantly (*p*-adj < 0.05) up or downregulated in the Aβ42 strain upon FMN supplementation (hereafter referred to as FMN_Aβ42, Supplementary Data 3), compared to 389 genes in the control strain (hereafter referred to as FMN_control, Supplementary Fig. 14b and Supplementary Data 4). We next performed gene set analysis (GSA) on the significantly changed genes (*p*-adj < 0.05), wherein 53 and 22 gene sets were found to be significantly enriched in the FMN_Aβ42 and FMN_control strain respectively (*p*-adj < 0.05, Supplementary Fig. 15). Thirty genes were significantly differentially expressed in both FMN_Aβ42 and FMN_control (*p*-adj < 0.05, log_2_ Fold change ≤ −1 or ≥ 1), with 17 and 8, respectively, out of these 30 genes being related to metabolic process and ion transport (Supplementary Fig. 16). This is consistent with the expected role of FMN as a cofactor for flavoproteins, participating in carbohydrate, amino acid, fatty acid and ion metabolic pathways^46^.

**Fig. 6:**
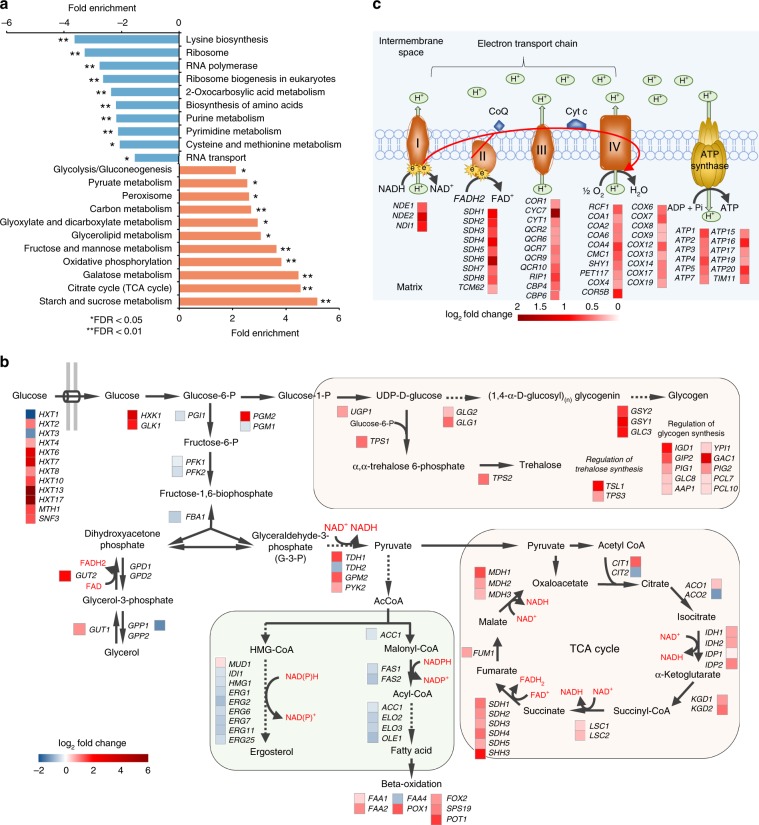
Transcriptional responses in Aβ42 strain with FMN supplementation. **a** Gene set enrichment analysis. Fold enrichment indicates the magnitude of enrichment in our dataset against the S288C yeast genome background using DAVID bioinformatics resources. **b** Fold changes in the expression of genes which encode central metabolic pathways. **c** Fold changes in expression of genes coding for mitochondrial electron transport chain proteins. All comparison is between FMN supplementation, and without (*p*-adj < 0.05 according to Benjamini–Hochberg method).

Gene set enrichment analysis also revealed that for the Aβ42 strain with FMN supplementation, pathways that were upregulated were mainly involved in carbohydrate metabolism, glycogen biosynthesis, ion transport and homeostasis, ATP biosynthesis, mitochondrial translation, and cellular response to oxidative stress. In contrast, gene sets related to ribosome function, cytoplasmic translation, and cellular amino acid biosynthesis were all downregulated in the Aβ42 strain with FMN supplementation (*p*-adj < 0.05, Fig. 6a and Supplementary Fig. 15a). Many genes involved in amino acid biosynthesis were also significantly downregulated in the Aβ42 strain with FMN supplementation (*p*-adj < 0.05, Supplementary Fig. 17). From these results one could speculate that FMN can improve protein homeostasis under stress by mediating the repression of amino acid biosynthesis and protein synthesis. This would enable a reduction in ER stress by reducing the load of newly synthesized proteins.

FMN supplementation significantly increased redox cofactors (Fig. 5a, b), that are mainly generated in carbohydrate metabolism^47^, so we further investigated the differentially expressed genes in central carbon metabolism (Fig. 6b). *HXT1*^48^ and *HXT3*^49^, encoding low affinity glucose transporters, showed to be downregulated in the Aβ42 strain with FMN supplementation. In contrast, genes encoding high affinity glucose transporters (*HXT2*, *HXT4*, *HXT6*, and *HXT7*), and hexose transporters (*HXT8*, *HXT10*, *HXT13*, and *HXT17*) were upregulated. Most genes (20/22) in the tricarboxylic acid (TCA) cycle were also upregulated after FMN supplementation as well (Fig. 6b). The TCA cycle generates NADH and FADH_2_, which in turn act as electron donors for oxidative phosphorylation in the electron transport chain. Meanwhile, we found that genes encoding respiratory complexes in the electron transport chain showed significantly (*p* < 0.05) increased transcription after FMN supplementation (Fig. 6c). Iron transport plays a vital role in living organisms by participating in a variety of electron transfer reactions and enzymatic reactions. Here, genes involved in iron transport and homeostasis were also mostly upregulated after FMN supplementation (Supplementary Fig. 18). In terms of what decreased in expression after FMN supplementation, genes involved in ergosterol and fatty acid synthesis, the second branch of pyruvate metabolism, were mainly downregulated, along with a reduced NADPH consumption to NADP^+^. This finding is consistent with the increased NADPH/(NADPH + NADP^+^) ratio we found in the FMN supplemented strains (Fig. 5a). The synthesis pathways for the storage carbohydrates glycogen and trehalose were also enriched with upregulated genes after FMN supplementation (Fig. 6b). The upregulated genes in trehalose synthesis may therefore link the stress resistant phenotype of FMN supplemented cells to the alternative role of trehalose as a stress protectant. Indeed, it has been reported previously that trehalose can stabilize the structures and enzymatic activities of proteins against thermal denaturation^50^, and can prevent the formation of Aβ aggregates and reduce its cytotoxicity^51^.

Our previous observations showed that Aβ42 expression induces strong ER stress and activates the UPR as a means of the cell attempting to reduce the misfolded protein burden generated by Aβ42^23^. After FMN supplementation of the Aβ42 strain, genes related to the oxidative stress response, protein degradation, and endocytosis were found to be significantly (*p* < 0.05) upregulated (Supplementary Fig. 19), suggesting cells are restoring ER homeostasis. These findings are consistent with western blot analysis showing that FMN supplementation decreased levels of Aβ42 aggregates (Fig. 4b).

### Overexpression of flavoprotein Dus2p reduces Aβ42 toxicity

FMN participates in many biological processes, as can be seen through our transcriptional analysis following FMN supplementation. This could principally be due to its role as an essential cofactor for a variety of FMN-dependent enzymes. To investigate the potential roles of flavoenzymes in general in Aβ42 toxicity, 11 strains with single flavoenzyme gene deletions were assessed. These flavoenzymes are involved in myriad roles, from reactions involving electron transfer in the mitochondria (Cyb2p, Glt1p, Pst2p), redox processes in the cytoplasm (Lot6p, Oye2p, Rfs1p), tRNA-modification (Dus2p, Dus3p, Dus4p), to central metabolic functions (Met5p, Aro2p)^52^. First, the 11 single deletion strains were transformed with a plasmid for constitutive expression of Aβ42. Thereafter, cellular viability during chronological aging was tested with 5 mM FMN supplementation. As found previously (Fig. 3e), we observed that FMN significantly increased Aβ42 survival. As in several strains, despite flavoenzyme genes being deleted, FMN supplementation was still found to significantly increase Aβ42 viability in the mutant strains: *rfs1∆*, *pst2∆*, *lot6∆*, *oye2∆*, *aro2∆*, *dus3∆* and *dus2∆* (*p* < 0.05). FMN supplementation however did not affect Aβ42 viability in *dus4∆*, *cyb2∆* and *glt1∆* mutant strains (*p* > 0.05). Finally, in contrast, FMN supplementation significantly decreased Aβ42 viability in the *met5∆* mutant strain (*p* < 0.05, Supplementary Fig. 20a).

Since *cyb2∆*, *glt1∆*, and *met5∆* significantly altered the beneficial effects of FMN supplementation in the Aβ42 strain, we tested to see if the opposite would be true if Aβ42 toxicity could be alleviated by overexpression of these genes. Except for *cyb2*, *glt1* and *met5* genes, *dus2* gene was also tested due to *dus2∆* significantly decreased Aβ42 viability (*p* < 0.05, 9.2 ± 0.9% of live cells in *dus2∆* strain vs 89.3 ± 0.9% of the control Aβ42 strain). Only *DUS2* overexpression significantly increased viability in Aβ42 strain, showing 71.4 ± 4.5% and 44.3 ± 5.2% of live cells, in 1- and 4-day-old stationary phase cultures respectively, compared to 60.8 ± 5.5% and 31.1 ± 3.4% of the control Aβ42 strain (*p* < 0.05, Supplementary Fig. 20b). This would therefore suggest that *DUS2* could be involved in modulating Aβ42 toxicity. The mRNA expression levels of human ortholog *DUS2* were also significantly lower in cerebellum, visual cortex and prefrontal cortex of AD patients, compared to controls (Supplementary Fig. 21). Moreover it has been reported that Dus2 serves as an inhibitor in the regulation of PKR function^53^, an interferon-induced protein kinase. As the activation of PKR is known to be related to ER stress-induced apoptosis in AD^54^, *DUS2* could be investigated as an additional gene target alongside *FMN1* for reducing Aβ42 toxicity.

### FMN reduces HTT103QP and α-synuclein toxicity

Many neurodegenerative disorders share a common hallmark of aberrant protein aggregation. To test the specificity of FMN effects, we assessed its effect on two other proteins: HTT103QP and α-synuclein, using humanized yeast models.

**Fig. 7:**
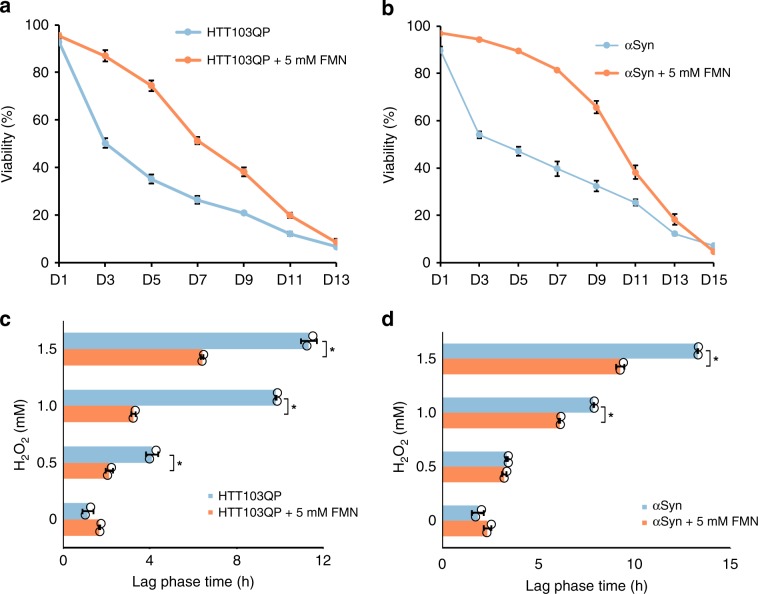
FMN supplementation reduces HTT103QP and α-synuclein toxicity. Viability of the HTT103QP strain (**a**) and the α-synuclein strain (**b**) with 5 mM FMN supplemented during chronological aging. Viability is shown as the fraction of PI negative cells (*n* = 3 ± SD). HTT103QP cells (**c**) and α-synuclein cells (**d**) display an increased H_2_O_2_ tolerance with FMN supplementation. Cells without or with FMN supplement were grown to OD ≈ 0.2 and treated with different concentrations of H_2_O_2_. Cell growth was monitored using a microplate reader with lag phase time analyzed using the R package growthrates. Results shown are average values ± SD of biological duplicates. The asterisk (*) indicates significant difference (*p* < 0.05). Source data are provided as a Source Data file.

HD develops when the CAG trinucleotide repeats cause the polyglutamine (polyQ) expansion in the Huntingtin (HTT) gene^55^. In yeast, repeat lengths of 47 or more lead to HTT protein aggregation and the formation of typical inclusion bodies that are similar to those of HD patients^56^. The HTT103QP model was reconstructed with 103 glutamines expansion under the control of constitutive *GPD* promoter. We then evaluated cellular viability in the presence of 5 mM FMN following chronological aging. The fractions of viable cells were significantly higher until day 11 in the HTT103QP strain with FMN supplementation, compared to without supplementation (Fig. 7a). In previous experiments, we observed that FMN supplementation was beneficial for oxidative stress tolerance in Aβ42 strain. Since increased oxidative stress has long been held as a key player in the HD pathogenesis^57^, we further investigated if FMN supplementation could protect HTT103QP cells from oxidative stress, as was the case in Aβ42 strain. The results showed that HTT103QP cells had prolonged lag phases during H_2_O_2_ stress, and the FMN supplementation could significantly shorten the extended lag phases (*p* < 0.05, Fig. 7c and Supplementary Fig. 22a).

The pathological hallmark of PD is the accumulation of high levels of oxidative free radicals and the presence of cytoplasmic inclusions called Lewy bodies, which predominantly composed of the misfolded and aggregated α-synuclein^58^. In the humanized PD yeast model, the expression of α-synuclein was controlled under the inducible *MET25* promoter, which is induced by depletion of methionine from the culture medium^59^. We observed that supplementation of FMN improved cellular viability in the α-synuclein strain as well (*p* < 0.05, Fig. 7b). The oxidative stress tolerance assay was also performed, which showed cells with FMN supplementation had a higher H_2_O_2_ tolerance than cultures without supplementation (*p* < 0.05, Fig. 7d and Supplementary Fig. 22b). These results indicate that FMN supplementation could offer increased oxidative stress tolerance in different neurodegenerative disease models.

## Discussion

In this study, we used genome-wide SGA screening to identify modulators of Aβ42 cytotoxicity. From nearly 5,500 mutants we identified a subset with increased sensitivity towards Aβ42, which were significantly enriched in protein secretion and degradation processes, as well as to cytoprotective mechanisms for maintaining cellular proteostasis. Moreover, we specifically identified riboflavin kinase (*FMN1* gene), and its metabolic product FMN, as critical modulators of Aβ42 toxicity. Transcription of *RFK*, the human orthologue of *FMN1*, was also found to be significantly decreased in brain tissues of AD patients (Fig. 2c), thereby suggesting a conserved evolutionary function of riboflavin kinase in underlying processes that govern proteostasis management in cells.

Brain cells are particularly susceptible to oxidative stress due to their higher metabolic activity and lower antioxidant activity, compared to many cells of other organs, suggesting that redox metabolism may be an important factor for neuronal death. There are several plausible models proposing how riboflavin could protect cells against oxidative stress by: (1) enhancing the glutathione redox cycle^60^, (2) deactivating the lipid peroxides^61^, (3) direct activity against free radicals^62^, and (4) enhancing antioxidant enzyme activities^63^. Oxidative stress is also often accompanied by mitochondrial dysfunction, which may exacerbate disease progression^64^ and it was shown in several case studies that riboflavin administration in patients with mitochondrial diseases can lead to clinical improvement^65,66^.

The crucial roles of riboflavin are linked to its active forms FMN and FAD. With a tricyclic heteroaromatic isoalloxazine ring, FMN and FAD can reversibly accept and donate one or two electrons, with the majority of flavoenzymes catalyzing reduction-oxidation reactions in metabolic pathways. Transcriptomics analysis of our yeast AD model showed that the genes involved in TCA cycle and oxidative phosphorylation processes were significantly upregulated by FMN supplementation (Fig. 6), suggesting that FMN mediates an increase in the generation of ATP and NADH. In addition, we also observed significantly downregulated expression of genes related to ergosterol and fatty acid synthesis pathways, indicating possible increase in the release of reduced NADPH (Fig. 6).

Declining mitochondrial function is known to lead to abnormal energy metabolism, which plays an important role in mediating AD dysfunction and degeneration^67,68^. Mitochondrial dysfunction also often accompanies elevated ROS production, leading to oxidative stress that may exacerbate AD progression through oxidative damage to cellular structures, proteins, lipids and DNA^64^. In our previous report, aberrant fragmented mitochondrial structures and increased ROS levels in our Aβ42 model were indeed observed^23^. The redox couples NAD(P)H/NAD(P)^+^ are the major electron transfer carriers for reduction-oxidation in cells, and thus vital for diverse biochemical processes. During aging, decreased NAD(P)H and redox ratios could contribute to AD progression, which may be due to a decline in TCA cycle enzymes activites^69^. Studies in aging and AD-like neurons from nontransgenic and 3 × Tg-AD mice showed that adding an NAD(H) precursor, in order to stimulate a balanced redox defense is neuroprotective^70^. In accordance with previous findings, we observed significantly increased NADPH/(NADPH + NADP^+^) and NADH/(NADH + NAD^+^) ratios and cellular tolerance to oxidative stress in our Aβ42 strain when supplemented with FMN (Fig. 5), which may partially contribute to the increased viability during chronological aging (Fig. 3e, f and Supplementary Fig. 11). Targeting redox metabolism and FMN cofactors may thereof be an effective therapy for combating oxidative stress in aging and AD.

Another important group of flavoenzymes are involved in reactions related to tRNA-modifications. In particular, the *Dus1-4* family of FMN-dependent flavoenzymes, encode tRNA-dihydrouridine synthases, which reduce uracil to dihydrouridine at specific positions on tRNA. Moreover, this is one of the most common modifications of nucleosides in tRNA in all living cells^71^. So far, only one dihydrouridine synthase in human has been identified, which is an ortholog of the yeast Dus2p^72^. The human Dus2p has also been found to be upregulated in pulmonary carcinogenesis patients, resulting in higher levels of dihydrouridine modification^73^. In our Aβ42 strain, *dus2∆* significantly decreased cellular viability (Supplementary Fig. 20a), whilst *DUS2* overexpression significantly increased viability, suggesting that Dus2p might be a direct contributing factor towards Aβ42 detoxification (Supplementary Fig. 20b). Further investigation on the biological significance of Dus2 could improve our understanding of this flavoprotein in AD pathogenesis as well as help determine its potential as a therapeutic target against the disease.

With respect to the *fmn1* mutant identified from SGA screening, the lack of the functional enzyme resulted in the formation of multiple amorphous-like aggregates, rather than one or two large inclusions (Fig. 4a). Previous work has found these amorphous aggregates to be severely toxic to yeast cells^74^, however, supplementation of FMN, the product of *FMN1* activity, helped alleviate misfolded protein stress in control strain (Fig. 4c, d).

In AD, ER stress plays one of the principle roles in determining disease pathogenesis, as it leads to activation of the UPR, which aims to restore ER homeostasis. Previous findings show that UPR signaling can be modulated through finetuning the expression of various gene targets, depending on the nature of the stress^75^. In our previous study we found that Aβ40 triggers a mild stress response, whilst Aβ42 triggers a significantly stronger stress response, alongside a stronger UPR response, affecting many aspects of cell physiology^23^. Considering the global effects of Aβ42, we used genome-wide transcriptional analysis to understand how Aβ42 toxicity is alleviated by FMN supplementation. FMN showed to enhance the efficacy of UPR in the Aβ42 strain by downregulating amino acid synthesis and upregulating protein degradation (Supplementary Fig. 17 and 19). This may also explain our findings that Aβ42 aggregates in our Aβ42 strain were reduced following supplementation with FMN (Fig. 4b).

In the ER, the relative higher concentration of oxidized glutathione (GSSG) is essential for the formation of native disulphide bonds in nascent proteins^76^. Reduced glutathione (GSH) also contributes towards robust cell function by acting as an endogenous antioxidant and deactivating ROS. To control ER redox homeostasis during protein folding, oxidized GSSG should therefore be reduced to GSH through glutathione reductase, which requires FAD that is produced from FMN, as a coenzyme^77^. The connection between the glutathione cycle and its dependency on flavins in the ER, may therefore also contribute to the beneficial effects of FMN supplementation.

Genome-wide screenings have been performed in yeast models with a mutant HTT fragment or α-synuclein, which showed the toxicity medicated by these two proteins was regulated by different sets of conserved genes and pathways^78^. Genes that modify the mutant HTT fragment cluster in the cellular processes related to stress response, protein folding, and ubiquitin-dependent protein catabolism, whereas genes in the functional categories of lipid metabolism and vesicle-mediated transport processes are isolated in the α-synuclein screen^78^. A yeast genome-wide screen based on high content microscopy (HCM) found that huntingtin exon 1 (Httex1)-103Q expression showed the ribosome quality control (RQC) machinery as playing a key role in regulating the nuclear localization and adverse effects of Httex1-103Q proteins^79^. Although various biological processes were involved in AD, HD and PD, the oxidative stress is critical to neuronal death in neurological disorders^80^. Neurons are particularly vulnerable to oxidative damage due to their high oxygen consumption (20% of total consumption), abundance of peroxidation-susceptible lipid bilayer and weak antioxidant defense^80^. The beneficial effects of FMN for oxidative stress tolerance in AD, PD and HD yeast models showed potential to further explore the effectiveness in attenuating neurodegenerative pathology.

In summary, our Aβ yeast model provides a useful tool for identifying genetic modifiers of Aβ cytotoxicity and investigating their mechanisms of action. Furthermore, this study provides highly informative data regarding the beneficial effects of FMN supplementation on cellular metabolic activity, redox homeostasis and proteostasis in the Aβ42 strain. Due to a strong correlation between oxidative stress, metabolic syndrome and AD^81^, understanding how FMN and flavoenzymes attenuate Aβ-induced cytotoxicity could be highly beneficial for clarifying molecular mechanisms in AD onset and progression.

## Methods

### Plasmids and strains

All plasmids and yeast strains used in this study are specified in Supplementary Tables 5 and Table 6, respectively. The primers used in this study are listed in Supplementary Table 7. *E. coli* DH5α was used for plasmid amplification. Plasmids for gene overexpression were constructed by Gibson assembly of the gene fragments, which were amplified from CEN.PK 113-11C genomic DNA, with a p416 GPD vector backbone, resulting in plasmids p416 GPD-FMN1, p416 GPD-CYB2, p416 GPD-GLT1, p416 GPD-MET5 and p416 GPD-DUS2, respectively. Codon optimized human RFK gene was synthesized and digested with restriction enzymes and ligated into the p416 GPD plasmid. The Molecular Barcoded Yeast (MoBY) plasmids and empty vector control (p5586) were kindly provided by the Department of Chemistry & Molecular Biology, Gothenburg university^82^. Genomic integration was done by all-in-one plasmids pECAS9-gRNA-KanMX-XI-3 and pECAS9-gRNA-KanMX-XII-5, which express high-fidelity version of Cas9 nuclease and guide RNA for XI-3 and XII-5 site respectively^83^. The Cas9 cassette and vector backbone were amplified from pECAS9-gRNA-KanMX-tHFD1 plasmid using primers CAS9-L1/CAS9-R1 and CAS9-L2/CAS9-R2, respectively. The specific guide RNAs were selected by comparing all potential off-targets in the CEN.PK 113-7D genome using the online CRISPRdirect tool (http://crispr.dbcls.jp/)^84^. The pECAS9-gRNA-KanMX-XI-3 and pECAS9-gRNA-KanMX-XII-5 plasmids were generated by Gibson Assembly Master Mix (New England BioLabs). The pYES2 vector expressing GFP-tagged HTT103QP construct under the GAL1 promoter was described previously^85^. The HTT103QP and GFP fragment were amplified and assembled into p416 GPD vector backbone by Gibson assembly, resulting in p416 GPD-HTT103QP plasmid. The pUG23 α-syn plasmid expressing a full-length α-synuclein-EGFP sequence was kindly provided by Prof. Joris Winderickx, KU Leuven, Belgium^59^. The standard LiAC/SS carrier DNA/PEG method was used for yeast transformation^86^.

Control and Aβ42 strains were query strains for SGA screening, which were constructed through homologous recombination by co-transformation of a repair fragment and pECA9-gRNA plasmid to Y7092 strain (Supplementary Fig. 1). Two repair fragments, XI-3up + (GPDp-Aβ42-CYC1t) + (TEF1p-natNT2-ADH1t) + XI-3down and XII-5up + (GPDp-Aβ42-CYC1t) + (TEF1p-hphNT1-ADH1t) + XII-5down, were prepared for two copies of Aβ42 gene integration. The upstream and downstream homologous arms XI-3up and XI-3down were amplified from Y7092 genomic DNA with primers XI-3-up-L1/ XI-3-up-R1 and XI-3-down-L/ XI-3-down-R, respectively. The GPDp-Aβ42-CYC1t, TEF1p-natNT2-ADH1t and TEF1p-hphNT1-ADH1t cassettes were amplified from p416 GPD-Aβ42, pFA6a-natNT2 and pFA6a-hphNT1 plasmids, respectively. The upstream and downstream homologous arms XII-5up and XII-5down were amplified from Y7092 genomic DNA with primers XII-5-up-L/XII-5-up-R and XII-5-down-L2/ XII-5-down-R, respectively. The repair fragments were assembled by fusing the different DNA parts with overlapping PCR. For the control strain, the GPDp-CYC1t cassette was amplified from p416 GPD plasmid. Transformants were selected on YPD plate supplemented with 100 mg L^−1^ clonNat (Werner BioAgents) and 300 mg L^−1^ hygromycin B (Formedium). Control (CEN) and Aβ42 (CEN) strains used for verification were constructed in CEN.PK 113-11 C background following the same method. The homologous recombination-based method was used for the construction of a *fmn1* Hsp104-GFP strain. The Hsp104-GFP-Leu fragment along with 500 bp upstream sequence and 600 bp downstream sequence was amplified from WT Hsp104-GFP strain (BY4741 background) genomic DNA and transformed into *fmn1* mutant strain to replace endogenous *HSP104* sequence. SD-Leu plates containing 200 mg L^−1^ G418 (Formedium) were used for selection of transformants at 22 °C.

### Culture conditions

*E. coli* was grown in LB medium supplemented with 100 mg L^−1^ ampicillin or 50 mg L^−1^ kanamycin at 37 °C. Yeast strains were grown in YPD medium, Delft medium, SD-Ura medium or SD-Leu medium according to the auxotrophic and antibiotic phenotypes of the cells (Supplementary Table 6). YPD medium contained 10 g L^−1^ yeast extract, 20 g L^−1^ peptone, and 20 g L^−1^ glucose. The recipe of Delft medium was described previously^87^. 100 mg L^−1^ histidine and/or 100 mg L^−1^ uracil were supplemented when needed. The SD-Ura medium contains 6.7 g L^−1^ yeast nitrogen base without amino acids (YNB, Formedium), 770 mg L^−1^ complete supplement mixture without uracil (CSM-URA, Formedium) and 20 g L^−1^ glucose. For SD-Leu medium, 770 mg L^−1^ CSM-URA in SD-Ura medium was replaced by 690 mg L^−1^ complete supplement mixture without leucine (CSM-LEU, Formedium). Cells with the *MET25* controlled expression cassette for α-synuclein-EGFP were cultured in Delft medium with 1 mM methionine. The α-synuclein-EGFP expression was induced by depletion of methionine from the medium. Yeast cells were cultivated at 22 °C for essential temperature sensitive alleles or 30 °C if not specified. Cell density (OD_600nm_) was measured using a GENESYS20 spectrophotometry (Thermo Fisher Scientific).

### SGA analysis

The *S.cerevisiae* strain Y7092 was used as the SGA starting strain. Query strains were constructed by chromosomally integrating two copies of GPDp-Aβ42-CYC1t fragment (sample) with the antibiotic resistance genes (clonNat and hygromycin B) into Y7092 genome. The yeast single gene knock-out collection (SGA-V2) and the essential gene temperature sensitive allele collection (ts-V6) were kindly provided by Prof. Charlie Boone, University of Toronto, Canada. Thereafter, SGA mating was performed in biological duplicate as previously described^28^ to introduce the Aβ42 cassettes into the SGA-V2 and ts-V6 collections. A control set of the same collections was used as a control for the toxicity assay, which were constructed by introducing two copies of the GPDp-CYC1t fragment with the same antibiotic resistance genes. All the pinning steps for collection handling were performed using a SINGER ROTOR HDA Robot (Singer Instrument). To define genetic interactions, colony sizes of mutants with Aβ42 expression were quantified from images of final selection plates, and fitness scores relative to mutants with control were calculated via online SGAtools (http://sgatools.ccbr.utoronto.ca/)^88^. A score of >0 represented positive interaction, i.e., increased colony size. While a score of <0 represented negative interaction, i.e., decreased colony size. Based on these scores, mutants with significantly (*p*-adj < 0.05) affected Aβ42 toxicity were analyzed for global genetic interactions via spatial analysis of functional enrichment (SAFE) package^30^. Adjusted *P* < 0.05 and scores ≤ −0.2 or ≥0.2 were set as thresholds to identify significantly affected mutants. Enrichment of GO terms in gene sets of interests was analyzed using the GOstats package^89^.

### Verification of the putative interactions

To confirm the phenotype observed in SGA screening, mutants that showed significant differences between Aβ42 and control were tested manually by random spore analyses and complementation assays. For Random spore analysis^28^, spores were first resuspended in 1 ml of sterile H_2_O, then 40, 80, 80, and 160 µl of resuspended cells were plated onto agar plates selecting for the haploid, the single gene mutation, the query-gene expression (Aβ42 or control), and both single gene mutation and query-gene expression, respectively. Plates were incubated at 30 °C for 2–3 days. Cell growth under the four cultural conditions were compared between Aβ42 and control. Mutants with the Aβ42 expression of which few or no haploids grew on plates were scored as synthetically lethal. Mutants with the Aβ42 expression of which less haploids grew on plates comparing to mutants with control were scored as synthetically sick. Mutants with the Aβ42 expression of which more haploids grew on plates compared to mutants with control were scored as synthetically positive. Complementation assays were performed with a selected group of mutants with Aβ42/control expression which were complemented with the corresponding MoBY plasmids or the empty control vector (p5586). Serial dilutions (10-fold) of each transformant were grown on SD-Ura agar plates at 30 °C for 2–3 days.

### Analysis of human brain gene expression data

We analyzed brain expression datasets from control (N) and AD cases (GN314, GN326, GN327 and GN328) deposited in the GeneNetwork website (http://www.genenetwork.org) to define the target gene expression and associate them with AD. The study enlisted approximately 400 AD cases and 170 controls matched for age, gender and post mortem interval. Three brain regions (cerebellum, visual cortex, and dorsolateral prefrontal cortex) from the same individuals were profiled on a custom-made Agilent 44 K microarray. The individuals were genotyped on two different platforms, the Illumina HumanHap650Y array and a custom Perlegen 300 K array. Differential expressed genes between control and AD cases were then identified using a Mann-Whitney test.

### NADP(H) and NAD(H) measurements

Enzymatic cycling assays to determine NADP(H) and NAD(H) levels were performed according to the NADP^+^/NADPH Quantification Kit (Sigma) and NAD^+^/NADH Quantification Kit (Sigma). Cells were grown in Delft + 100 mg L^−1^ histidine + 100 mg L^−1^ uracil medium, then 5 OD_600nm_ of cells were collected at later exponential (EX) phase (OD_600nm_ ≈ 2) and post-diauxic (PD) phases. Collected cells were immediately added to 20 ml of cold methanol (pre-chilled to −80 °C) to quench cellular metabolism. Cells were centrifuged at −10 °C for 4 min (4000*×g*), then supernatant was removed. Cell pellets were freeze-dried for 2 h and stored at −80 °C until measurement. The dried cell pellet was resuspended in 500 µl of extraction buffer (Sigma) and added to 0.2 g of prechilled glass beads (425–600 µm diameter, Sigma). Cells were lysed via four rounds of vortexing for 20 s at speed 6200 rpm on the Precellys Evolution Homogenizer (Bertin technologies) followed by incubation for 1 min on ice. The cell lysates were centrifuged at 14000*×g* for 1 min at 0 °C, and supernatant was used to measure NADH(H) and NAD(H) amounts following the manufacturer’s instructions (Sigma).

### H_2_O_2_ treatment

For growth assays, the Aβ42 strain was grown to mid exponential phase (OD_600nm_ ≈ 0.5–0.6) in Delft + 100 mg L^−1^ histidine + 100 mg L^−1^ uracil medium, the *HTT103QP* strain was grown to early exponential phase (OD_600nm_ ≈ 0.2) in Delft + 100 mg L^−1^ histidine medium, and the α-syn strain was grown to early exponential phase (OD_600nm_ ≈ 0.2) in Delft + 100 mg L^−1^ uracil medium, then 1 ml of cells were distributed on 48-well MTP flowerPlates (m2p-labs). Different concentrations of H_2_O_2_ were then added to the cells. Cell growth was monitored for biological duplicates using a BioLector reader (m2p-labs) and analyzed with R/growthrates package.

For H_2_O_2_ spotting tests, the control strain was grown to stationary phase. 1 OD_600nm_ of cells were pelleted and spotted at a series of 5-fold dilutions onto the Delft + 100 mg L^−1^ histidine + 100 mg L^−1^ uracil agar plates containing different concentrations of H_2_O_2_. Plates were then imaged after incubation at 30 °C for 2–3 days.

### Quantitative real-Time PCR (qPCR) analysis

qPCR was performed as previously described^90^. Aβ42 and control strains were grown in Delft medium supplemented with 100 mg L^−1^ histidine and 100 mg L^−1^ uracil. Samples were taken from biological triplicate cultures during exponential phase and after 1, 2, 3 days of cultivation. *ACT1* was used as a reference gene to normalize RNA levels with primers ACT-L/ACT-R. *FMN1* gene expression was measured with primers FMN1-L2/FMN1-R2. Fold changes were calculated according to *FMN1* gene expression in control strain and Aβ42 strain, respectively, during exponential phase.

### FMN and FAD supplementation

Riboflavin 5′-monophosphate sodium salt (FMN, Sigma) or riboflavin adenine dinucleotide disodium salt (FAD, Sigma) was prepared in distilled H_2_O to 70 mM stock solutions, which was filter-sterilized using 0.2 µm cellulose acetate filters (VWR). Yeast strains were grown overnight, diluted to an initial OD_600nm_ of 0.1 in Delft medium or SD-Ura medium. FMN stock solution was added to the culture to different final concentrations for continuous treatment. For agar plates, FMN and FAD stock solutions were added to the final concentration of 5 mM, respectively.

### Viability measurement

Viability was measured by propidium iodine (PI, Thermo Fisher Scientific) staining as described previously^23^. 0.5 OD_600nm_ of cells were taken at different culture ages (days) and stained with 0.5 µg ml^−1^ of PI in the dark at room temperature for 20 min. The cell pellet was washed twice with PBS and resuspended in 1 ml of PBS. Fluorescence was analyzed using a Guava flow cytometer (Merck). In all, 5000 cells were analyzed for each sample. Cells were also imaged under a fluorescence microscope (Leica DMI4000B) with DIC and RFP filters. Experiments were performed in biological triplicates.

### Inclusion body morphology test

WT Hsp104-GFP and *fmn1* Hsp104-GFP strains were grown to mid exponential phase (OD_600nm_ ≈ 0.5) in SD-Leu medium with G418 supplement and subjected to a heat shock at 38 °C for 90 min to induce protein aggregation. Samples were taken after 90 min of heat shock and fixed immediately in 3.7% formaldehyde. Cells were then imaged with a Carl Zeiss axiovert 200 M microscope (Zeiss). Images were processed with ImageJ (NIH), and at least 500 cells with aggregates were analyzed and quantified for each sample. Data is based on the average of six biological replicates.

### AZE treatment

L-azetidine-2-carboxylic acid (AZE), an analogue of proline, is incorporated competitively with proline into proteins during protein synthesis, and eventually causing conformational changes and protein misfolding^91^. The control strain was grown to mid exponential phase (OD_600nm_ ≈ 0.5–0.6) in Delft medium supplemented with 100 mg L^−1^ histidine and 100 mg L^−1^ uracil. AZE (Sigma) was added to the medium to final concentrations of 5 mM or 10 mM. After 3 h of cultivation at 30 °C, 0.2 OD_600nm_ of cells were pelleted and resuspended in 1 ml of sterile H_2_O. Serial dilutions (10^−1^, 10^−2^, 10^−3^) were used for spot tests on Delft + 100 mg L^−1^ histidine + 100 mg L^−1^ uracil agar plates. Viability was also determined by colony forming units (CFU) counting. In all, 400 cells were plated on Delft + histidine + uracil agar plates. Plates were incubated at 30 °C for 2–3 days. Results are from biological triplicates.

### Western blot

Aβ42 and control strains were grown to stationary phase in Delft medium supplemented with 100 mg L^−1^ histidine and 100 mg L^−1^ uracil. In all, 5 OD_600nm_ of cells were collected. Whole cell protein extraction and western blotting were carried out following previous protocols^21^. Aβ42 levels were identified by a mouse monoclonal anti-Aβ antibody (6E10, Covance, Catalog Number: SIG-39320, 1:2000 dilution). Anti-GAPDH (Santa Cruz, Catalog Number: sc-365062, 1:2000 dilution) was included as a loading control.

### Transcriptome profiling

Samples for RNA-seq analysis were taken from biological triplicates of cells grown until late EX phase (OD_600nm_ ≈ 2). In all, 10 OD_600nm_ of cells were collected in 50 ml falcon tubes containing 25 ml ice and snap-frozen in liquid nitrogen to prevent mRNA turnover and RNA extraction was performed as described previously^23^. RNA-seq was performed by the Novo Nordisk Foundation Center for Biosustainability of Technical University of Denmark (DTU). The Illumina TruSeq samples preparation kit v2, with poly-A enrichment, was used for RNA sequencing. The fragments were clustered on cBot and sequenced on a single lane on a HiSeq 4000 with paired ends (2 × 150 bp), according to the manufacturer’s instructions. The number of read pairs ranged from 12.0 to 16.0 million for each sample. Raw RNA-seq data was preprocessed by Nfcore-RNA-seq pipeline (https://github.com/nf-core/rnaseq), developed by the National Genomics Infrastructure at SciLifeLab, Karolinska Institute. The Report GO terms were performed using the Platform for Integrative Analysis of Omics (PIANO) R package^92^ with information from the Saccharomyces Genome Database (https://www.yeastgenome.org/). The functional enrichment of KEGG pathways was analyzed using the Database for Annotation, Visualization and Integrated Discovery (DAVID, https://david.ncifcrf.gov/)^93,94^. The differential gene expression (Log_2_FoldChange) and corresponding significance (*p*-adj) were calculated by the Benjamini–Hochberg method and used as input. Heatmaps of significantly changed GO terms and genes were generated by using pheatmap R package.

### Statistics

Significance of differences observed between strains were determined as mean ± SD using two-tailed student *t*-test. Three biological replicates were analyzed unless specified explicitly. Statistical significance was indicated as *p* < 0.05 unless specific explicitly.

### Reporting summary

Further information on research design is available in the Nature Research Reporting Summary linked to this article.

## Supplementary information

Supplementary Information

Description of Additional Supplementary Files

Supplementary Data 1

Supplementary Data 2

Supplementary Data 3

Supplementary Data 4

Reporting Summary

## Data Availability

The RNA-seq raw data can be downloaded from the Genome Expression Omnibus website (GEO, https://www.ncbi.nlm.nih.gov/geo/) with series number GSE128905. The data that support the findings of this study are available from the corresponding author upon reasonable request. The source data underlying Figs. 3c, e, 4a, b, d, 5, 7 and Supplementary Figs. 11a, 13a, 20, and 22 are provided as a Source Data file.

## Source data

Source Data
