# Supplementary material for: FMN reduces Amyloid-β toxicity in yeast by regulating redox status and cellular metabolism
Source: Nat Commun. 2020 Feb 13;11:867. doi: 10.1038/s41467-020-14525-4 (PMC7018843; doi:10.1038/s41467-020-14525-4)
Supplement: Supplementary file 1 — Supplementary Information [file 41467_2020_14525_MOESM1_ESM.pdf]

Supplementary Information

FMN reduces Amyloid-β toxicity in yeast by regulating redox status and cellular metabolism

Chen *et al*.

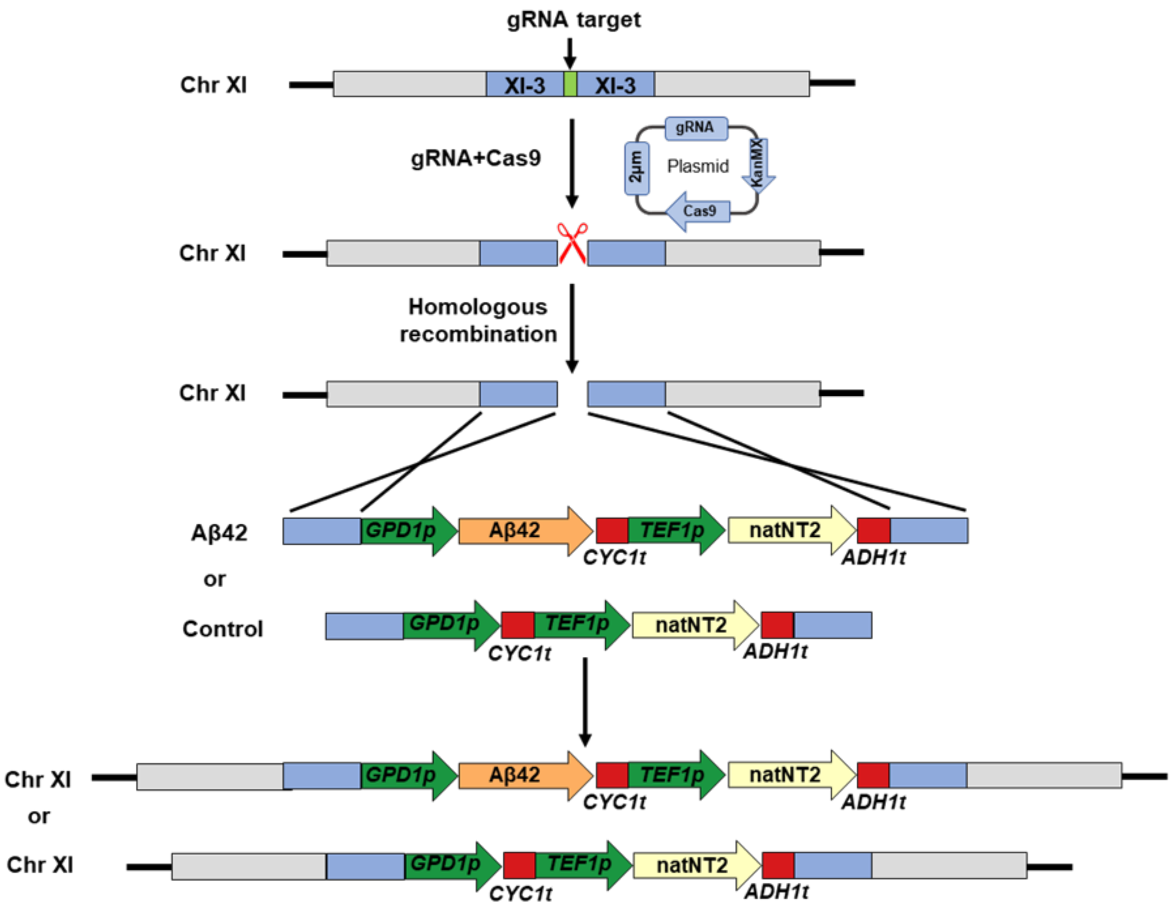

Supplementary Figure 1. The schematic diagram of SGA query strain construction. The first copy of Aβ42 gene under the control of *GPD1* promoter and *CYC1* terminator is integrated into chromosome XI of the yeast strain, Y7092. The all-in-one plasmids containing Cas9 nuclease and site-specific guide RNA are used for genome integration. The second copy of Aβ42 gene is integrated into chromosome XII (not shown). The control strain was constructed at the same chromosome sites with only promoter and terminator sequences. NatNT2 encodes clonNat protein as a selection marker.

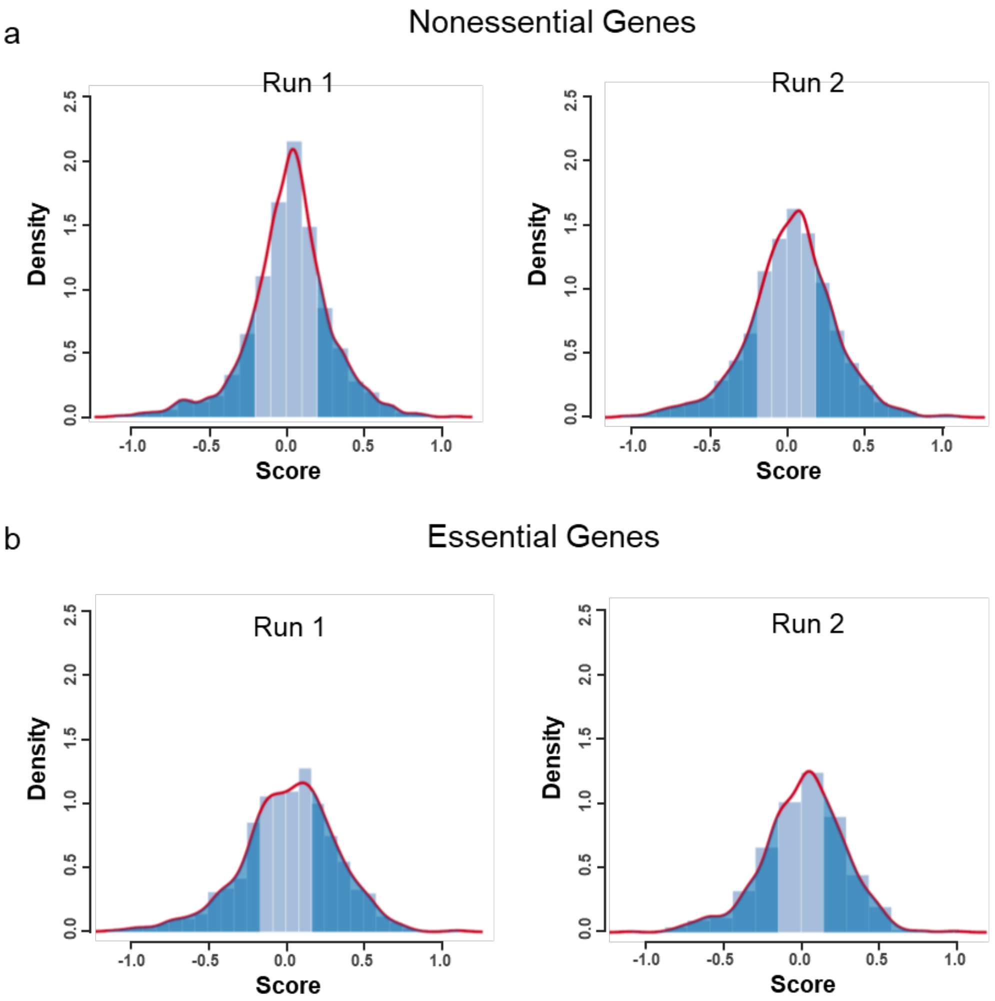

Supplementary Figure 2. The distribution of significantly changed SGA scores from the nonessential (a) and essential mutant collections (b) (*p*-adj < 0.05). Dark blue indicates the threshold set for the scores (score ≥ 0.2 or score ≤ -0.2).

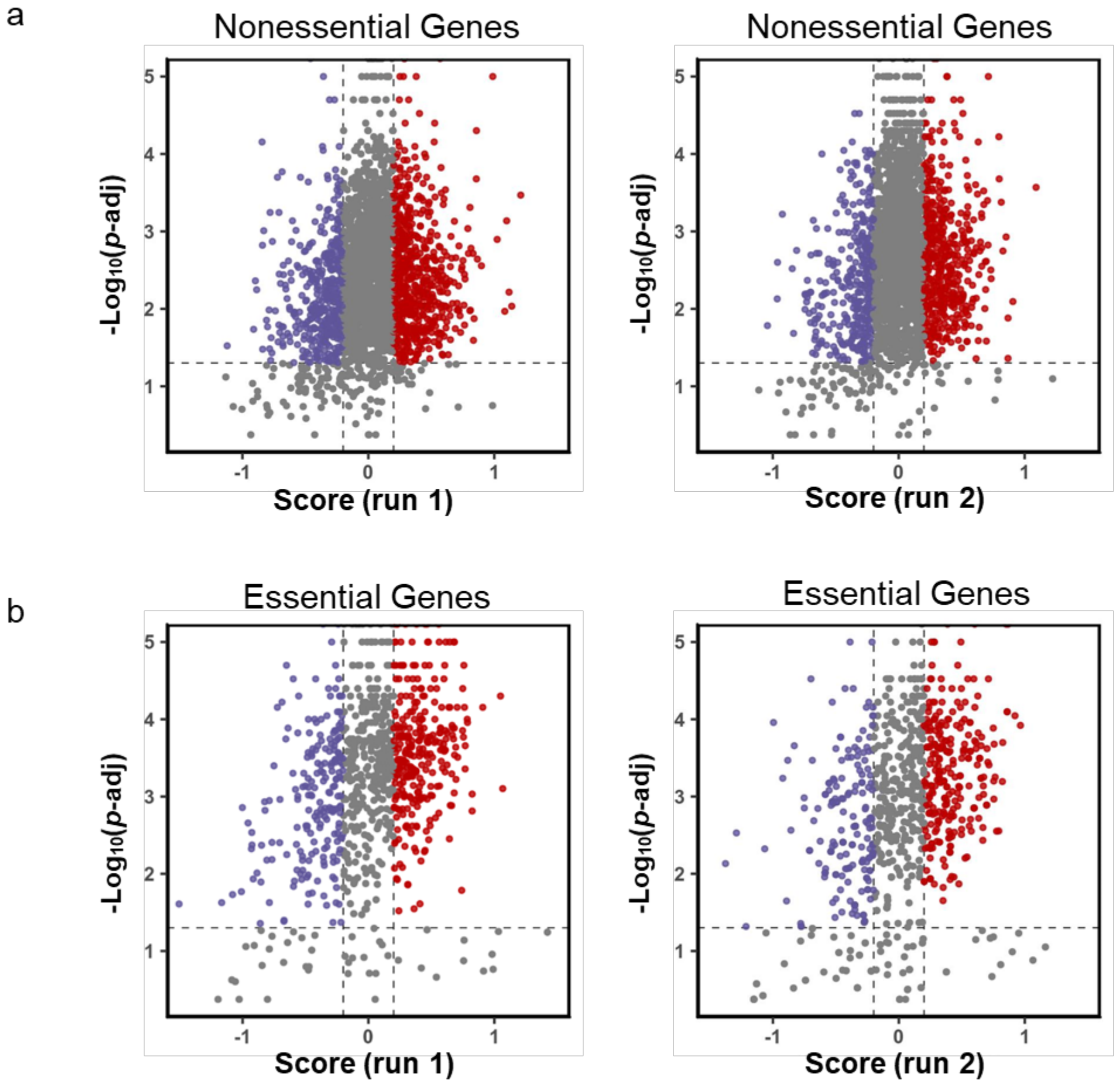

Supplementary Figure 3. Volcano plot of significantly changed SGA scores from the nonessential (a) and essential mutant collections (b). The SGA scores and corresponding significance values are shown on the X- and Y-axis, respectively. The mutants with significantly reduced Aβ42 toxicity (score ≥ 0.2, *p*-adj < 0.05) and significantly increased Aβ42 toxicity (score ≤ -0.2, *p*-adj < 0.05) are highlighted in red and blue, respectively. Adjusted *p* values (*p*-adj) are calculated using the Benjamini-Hochberg procedure.

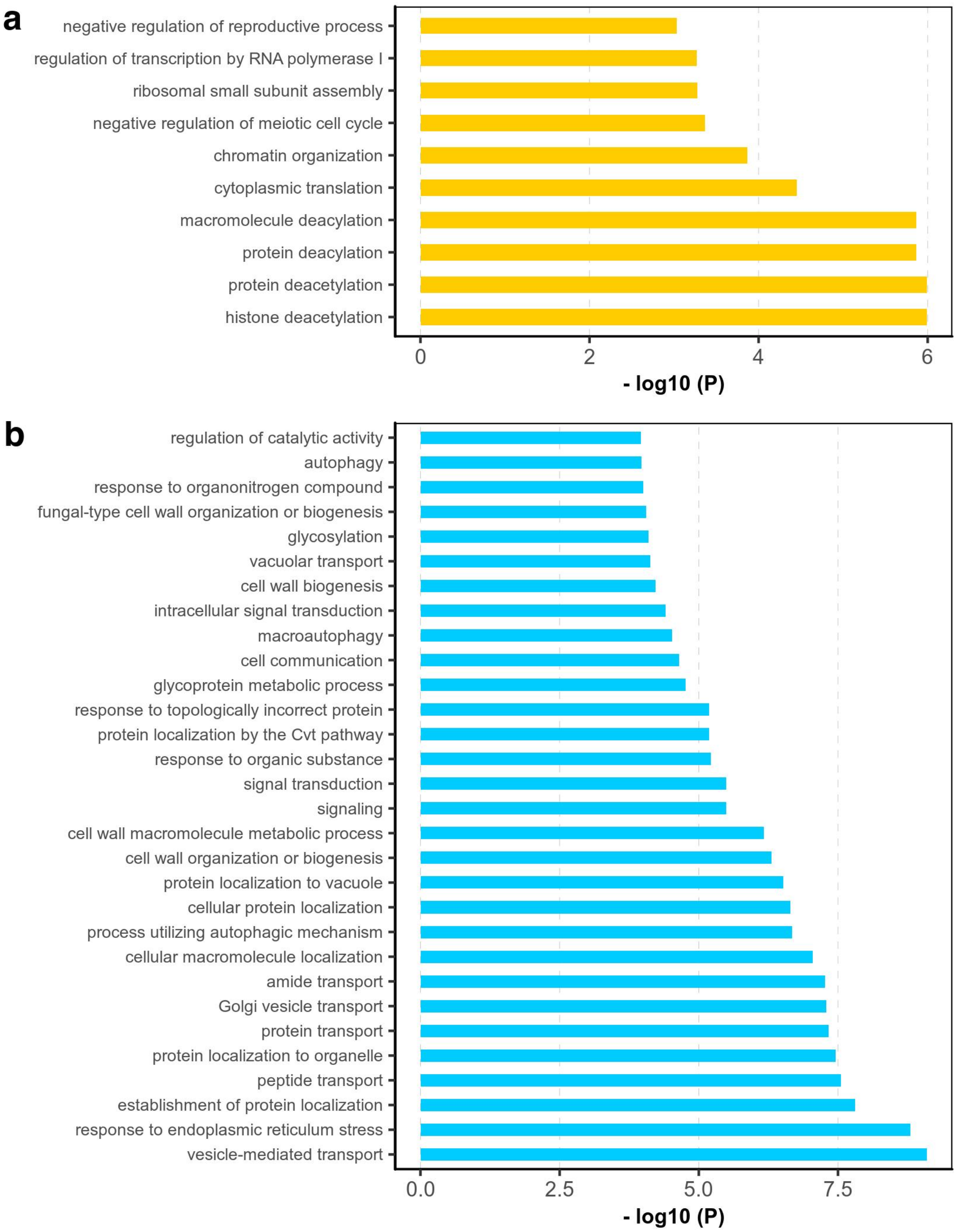

Supplementary Figure 4. Gene set enrichment analysis (GSEA) of mutants from the nonessential mutant collection with (a) significantly reduced Aβ42 toxicity (score ≥ 0.2, *p*-adj < 0.05) and (b) significantly increased Aβ42 toxicity (score ≤ -0.2, *p*-adj < 0.05). GO terms are ranked according to the significance and top 30 terms are listed in the figure.

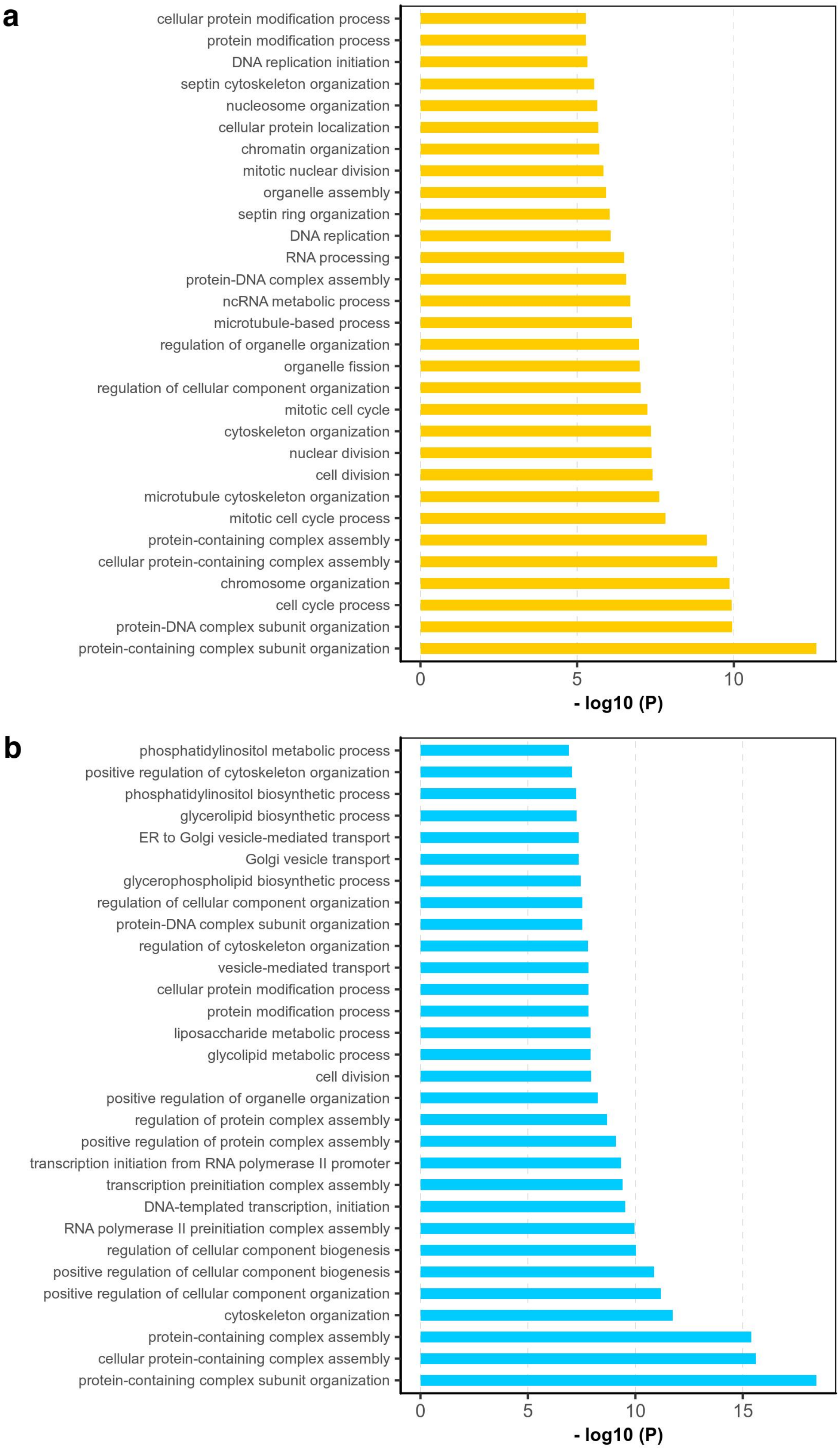

Supplementary Figure 5. Gene set enrichment analysis (GSEA) of mutants from the essential mutant collection with (a) significantly reduced Aβ42 toxicity (score ≥ 0.2, *p*-adj < 0.05) and (b) significantly increased Aβ42 toxicity (score ≤ -0.2, *p*-adj < 0.05). GO terms are ranked according to the significance and top 30 terms are listed in the figure.

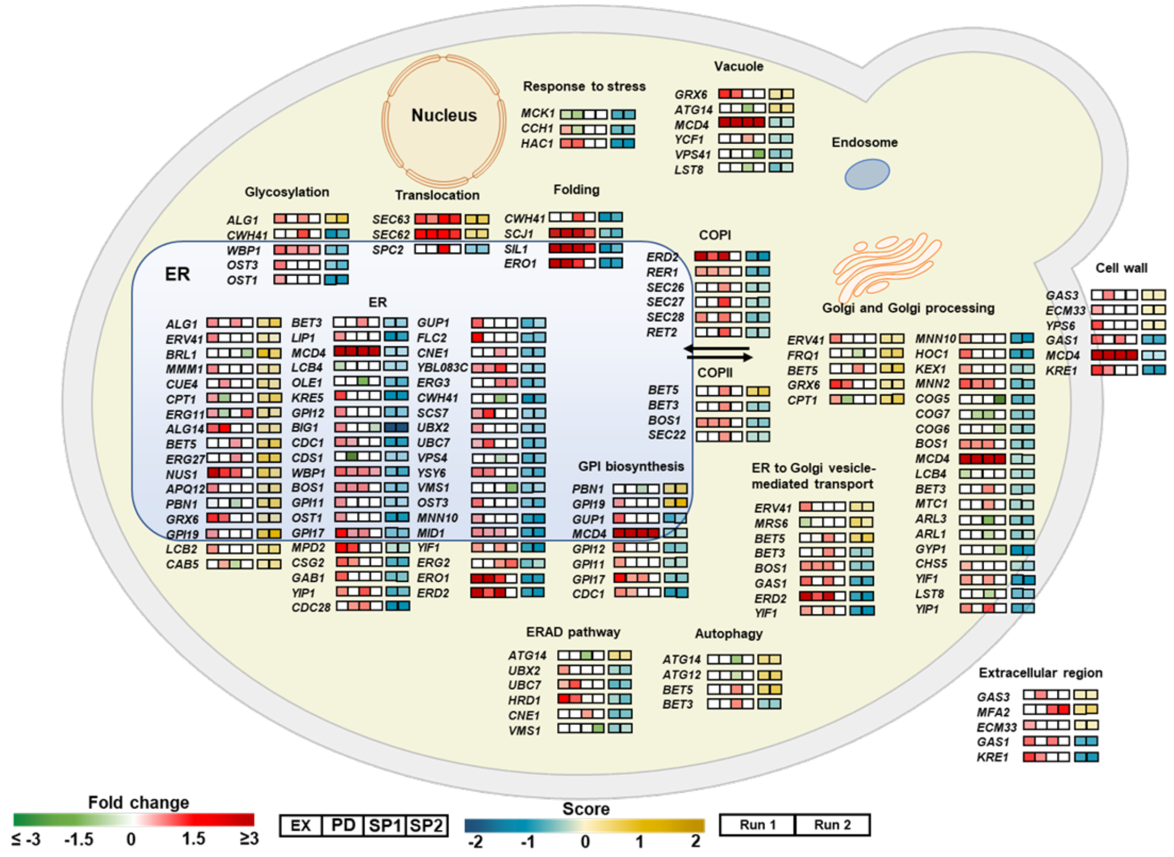

Supplementary Figure 6. The correlation between transcriptome and SGA data in protein secretion and degradation processes. Transcriptome data (in red and green) are shown as fold changes of gene expression between the Aβ42 expression strain and the control strain (carrying an empty vector) during EX, PD, SP1 (early stationary phase when extracellular carbon source was depleted) and SP2 (later stationary phase, 2 days after SP1) (Chen *et al*. 2017). SGA data (in blue and yellow) are shown as a score by comparing mutants with Aβ42 expression to mutants with the control from two independent screen (present study).

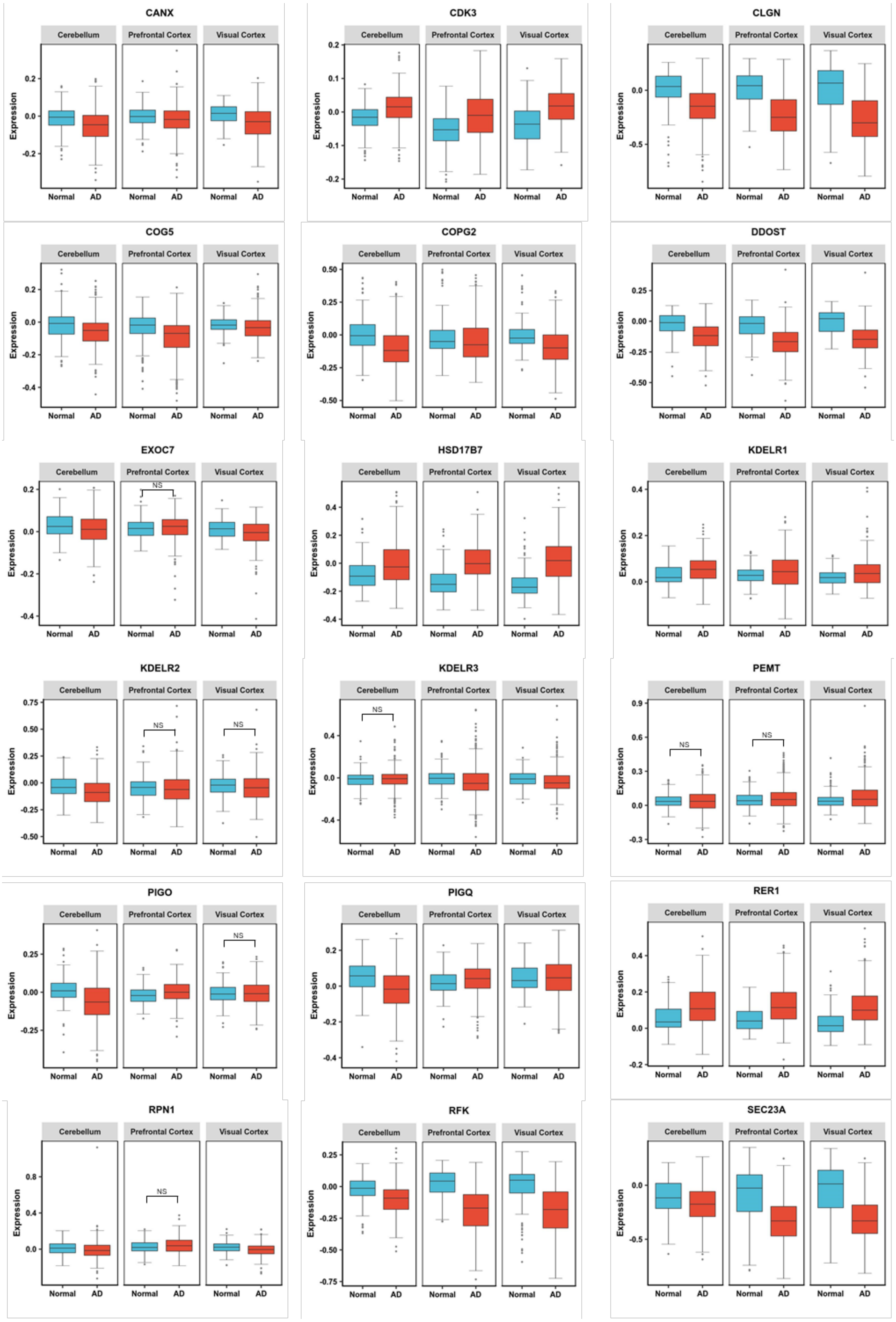
CANX
CDK3
CLGN
COG5
COPG2
DDOST
EXOC7
HSD17B7
KDELR1
KDELR2
KDELR3
PEMT
PIGO
PIGQ
RER1
RPN1
RFK
SEC23A
Cerebellum
Prefrontal Cortex
Visual Cortex
Expression
Normal
AD
NS

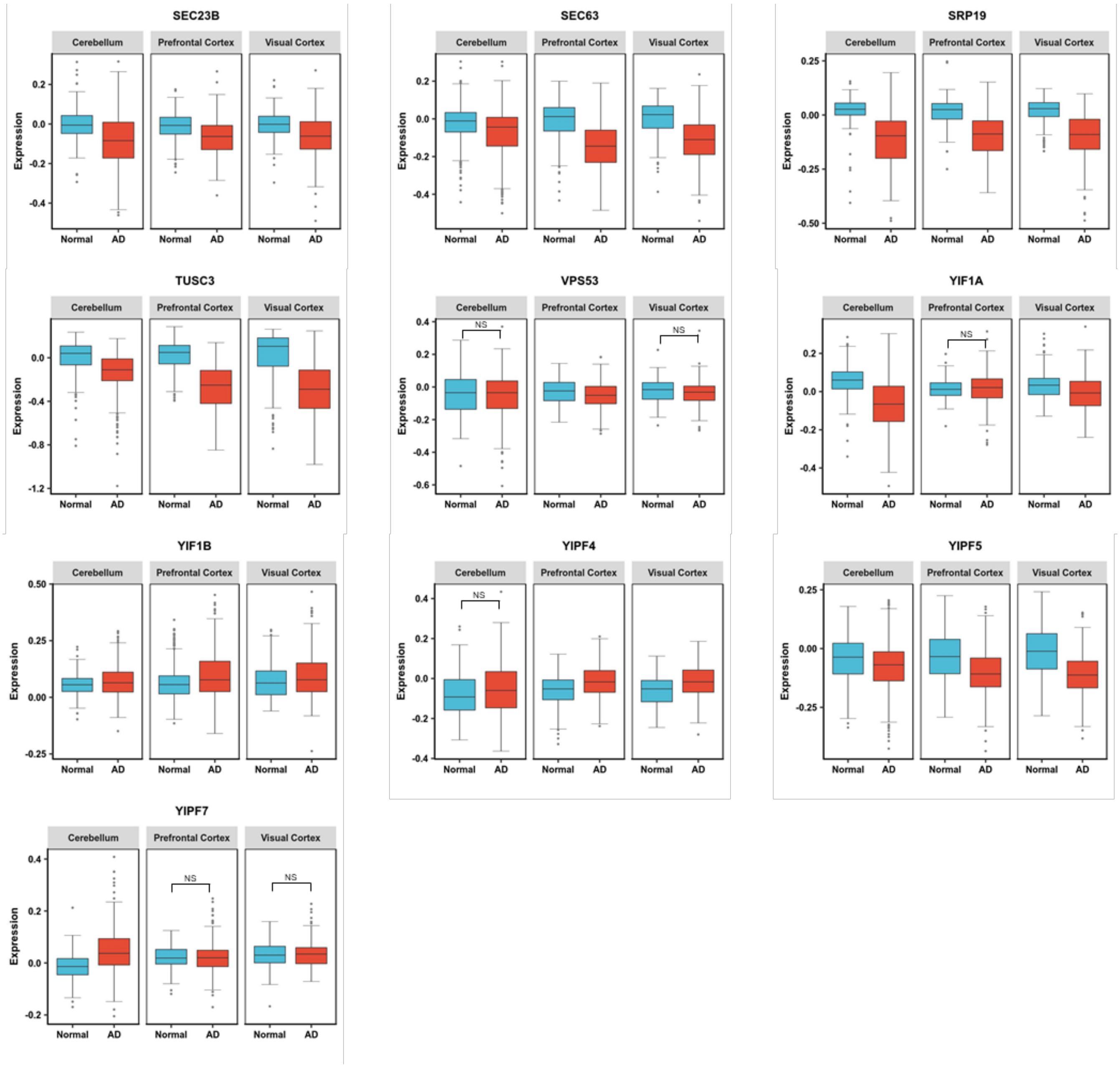

Supplementary Figure 7. The mRNA expression levels of 28 human orthologs in the cerebellum, prefrontal cortex and visual cortex of AD patients versus normal controls. NS indicates no significant difference (p-adj > 0.05) between AD patients and normal controls, whereas the remaining comparisons (without labeling) indicate significant difference (p-adj < 0.05).

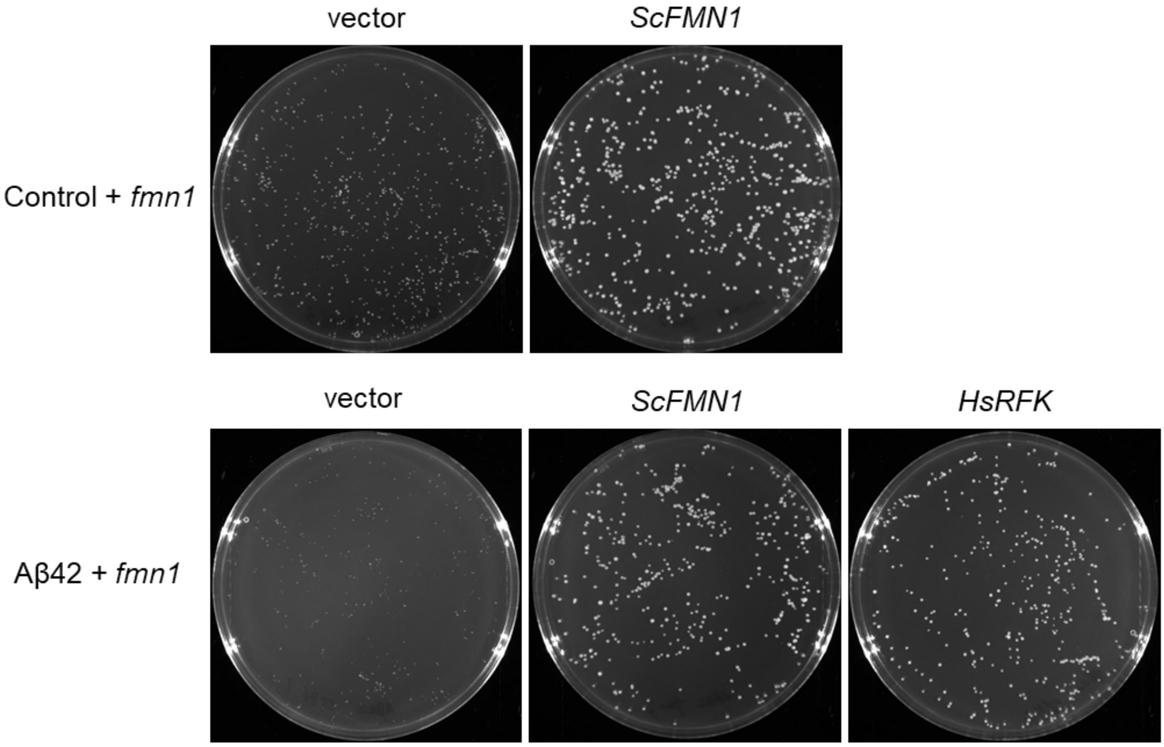

Supplementary Figure 8. Complementation assays of the *fmn1* mutant (in control or Aβ42 expressing strains) with either yeast (*Sc*) *FMN1* or human (*Hs*) homologues *RFK* expression. Cells are harvested at mid exponential phase (OD_{600nm} ≈ 0.5-0.6) and 400 cells are plated on selective plates. Vector: MoBY empty plasmid; *ScFMN1*: pFMN1-MoBY plasmid; *HsRFK*: P416 GPD-human RFK plasmid.

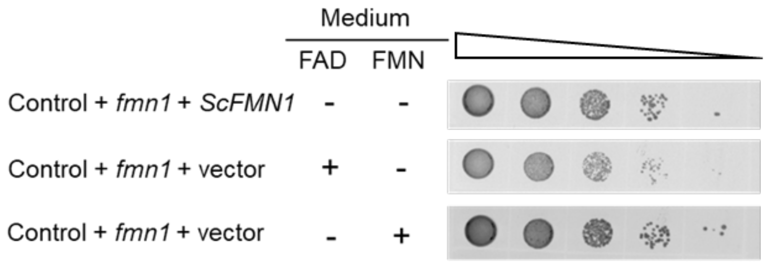

Supplementary Figure 9. 10-fold serial dilutions of the control strain with *fmn1* mutant expressing indicated constructs with the supplementation of 5 mM FAD or 5 mM FMN in the medium.

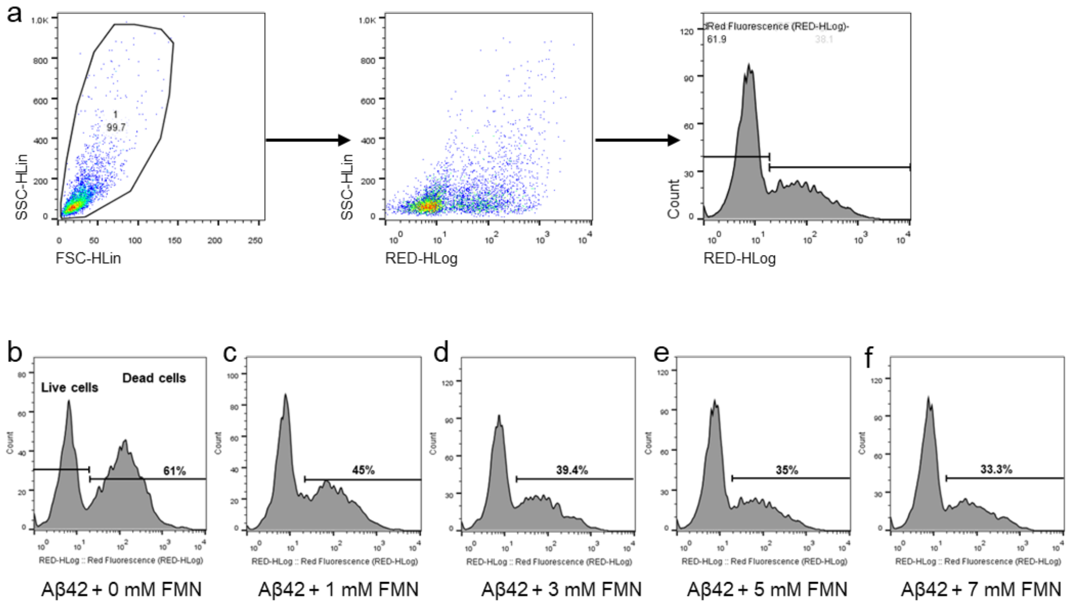

Supplementary Figure 10. Flowcytometry measurement of propidium iodide (PI) staining in the Aβ42 strain at day 5. (a) Gating strategy to detect live cells and dead cells. Aβ42 strain was treated without FMN (b), or with 1 mM FMN (c), 3 mM FMN (d), 5 mM FMN (e) and 7 mM FMN (f), respectively. The percentages of dead cells are annotated in the figure. Values shown are averages ± SD of biological triplicates. The same gating strategy was applied to all FACS results in this paper. Source data are provided as a Source Data file.

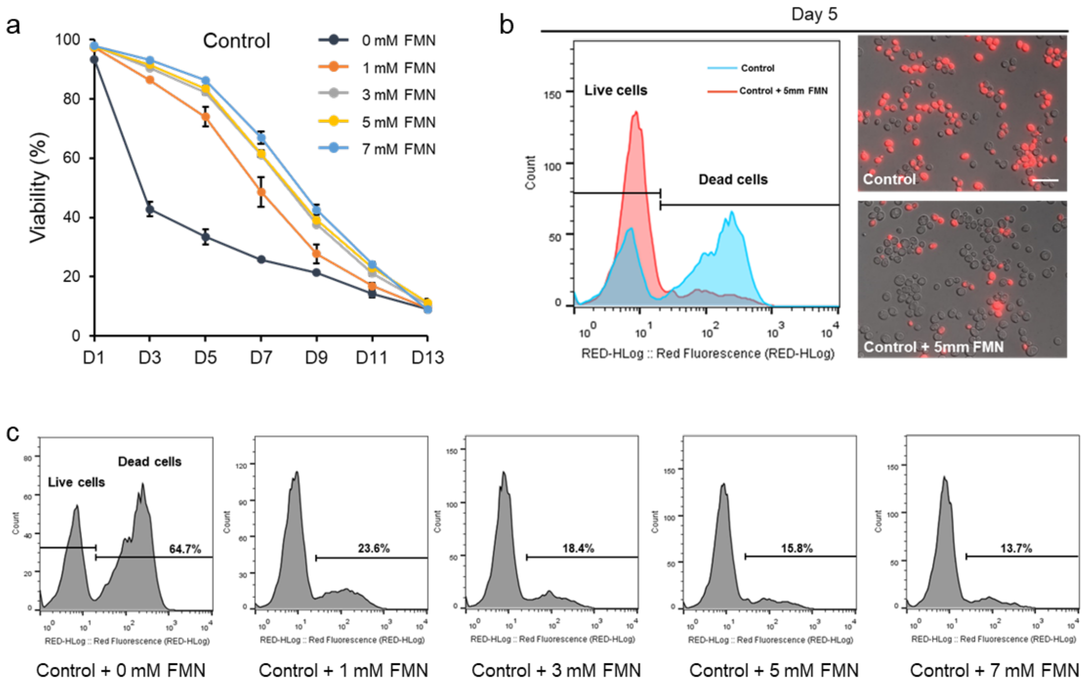

Supplementary Figure 11. Viability of control strain without or with different concentrations of FMN supplementation. (a) Viability was measured by PI staining following chronological aging. (b) Flowcytometry and fluorescence micrographs of PI staining on day 5. Dead cells are stained in red. Scale = 10 μm. (c-g) Flowcytometry measurement of PI staining in control strain at day 5. Cells was treated without FMN (c), or with 1 mM FMN (d), 3 mM FMN (e), 5 mM FMN (f) and 7 mM FMN (g), respectively. The percentages of dead cells are indicated in the figure. Values shown are averages ± SD of biological triplicates. Source data are provided as a Source Data file.

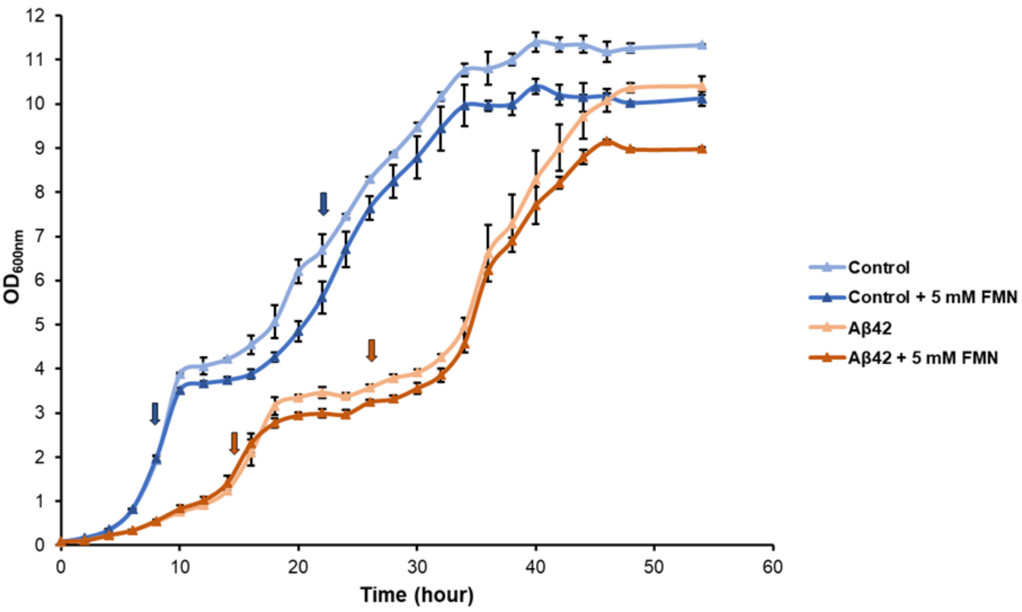

Supplementary Figure 12. Growth curve of control and Aβ42 strains without or with 5 mM FMN supplementation. Arrows indicate the sampling time points for NADPH/(NADPH+NADP$^+$) and NADH/(NADH+NAD$^+$) measurements during EX and PD growth phases. Arrows during EX growth phase also indicate the sampling time point for RNA-seq analysis. Values shown are averages ± SD of biological triplicates.

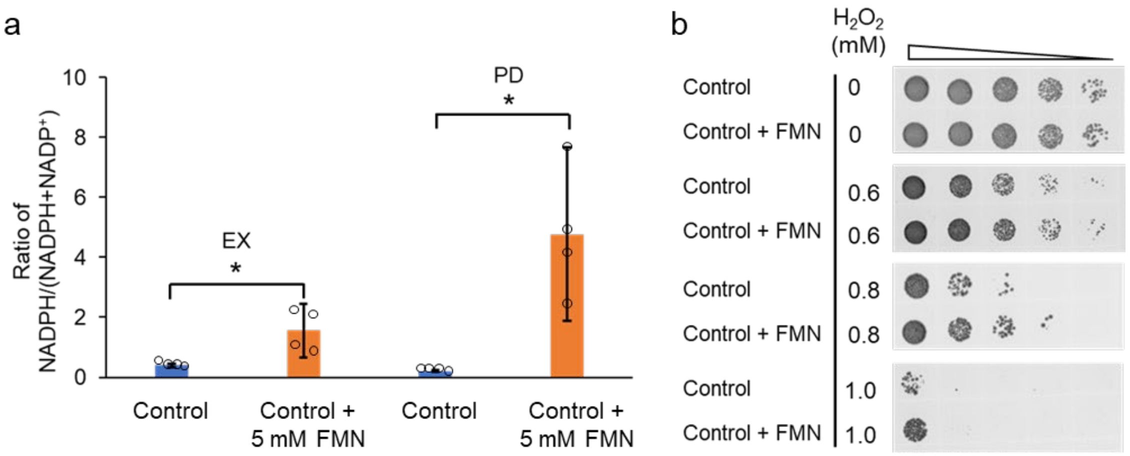

Supplementary Figure 13. FMN improves cellular tolerance to oxidative stress in the control strain. (a) The ratio of NADPH/(NADPH+NADP$^+$) in control strain without or with FMN supplementation. Results are presented as average values ± SD of biological quadruplicates. * = p < 0.05. (b) 5-fold serial dilutions of control strain without or with 5 mM FMN supplementation on plates containing 0.6 mM, 0.8 mM and 1.0 mM of H_2O_2, respectively. Source data are provided as a Source Data file.

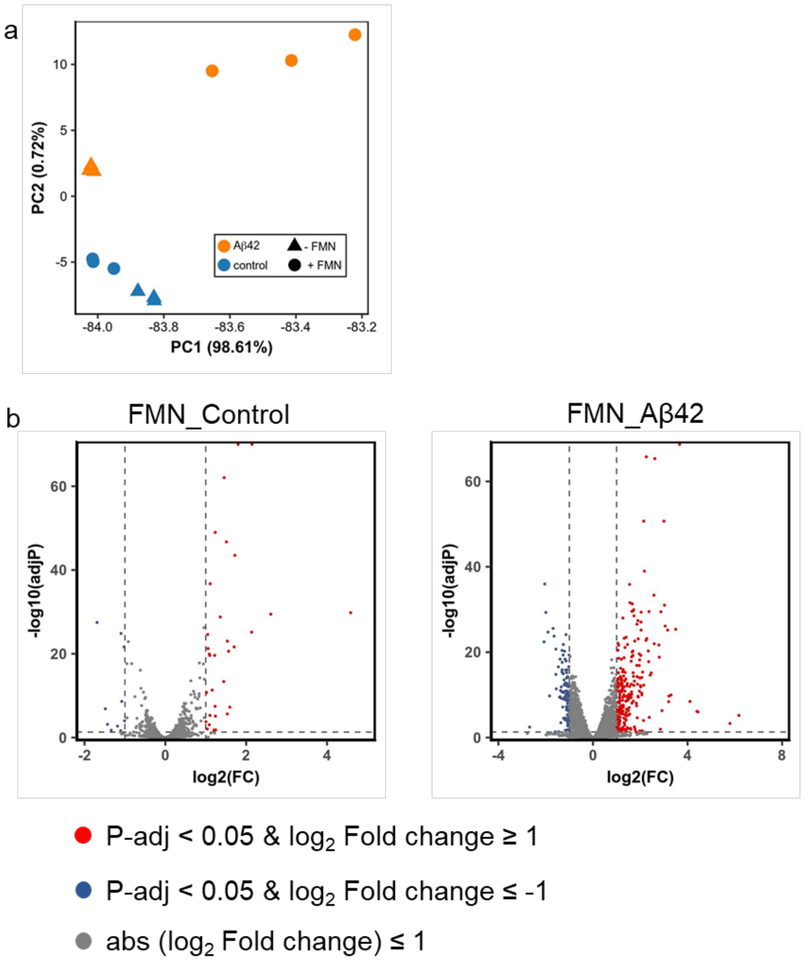

Supplementary Figure 14. The global transcriptional response to FMN supplementation in both the control and Aβ42 strains. (a) Principle Component Analysis (PCA) of the normalized RNAseq data. Samples were taken from biological triplicate cultures. (b) Volcano plot of $\log_2$ Fold change vs adjusted *p* value of differentially expressed genes comparing with FMN and without FMN supplementation in the control strain (left panel, FMN_Control) and Aβ42 strain (right panel, FMN_Aβ42). The dashed vertical grey line indicates the threshold of $\log_2$(Fold change) (≤ -1 or ≥ 1), while the horizontal grey line indicates statistical significance threshold of adjusted *p* value < 0.05.

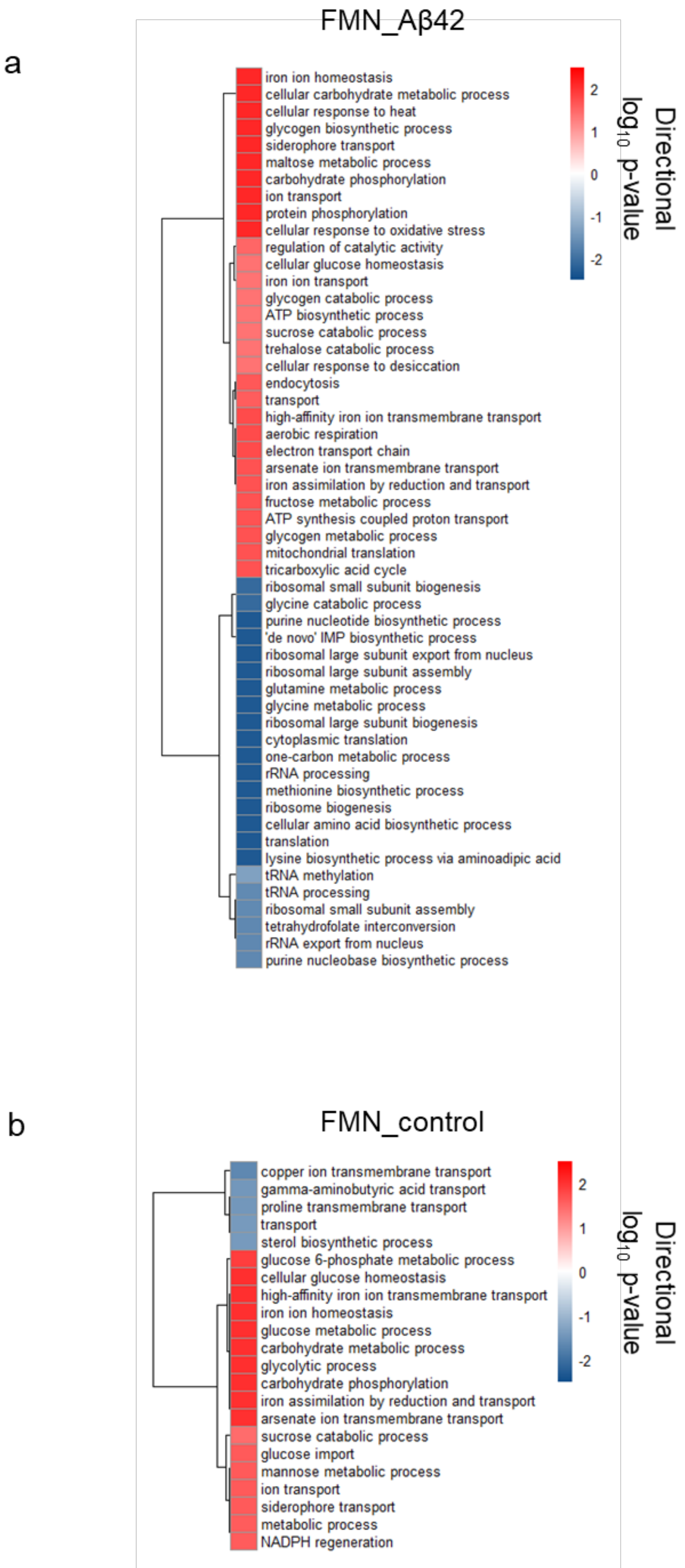

Supplementary Figure 15. GO enrichment analysis. Significantly enriched GO terms in Aβ42 strain (a) and control strain (b) with or without FMN supplementation. Up-regulated processes (red) and down-regulated processes (blue) are presented by their significance.

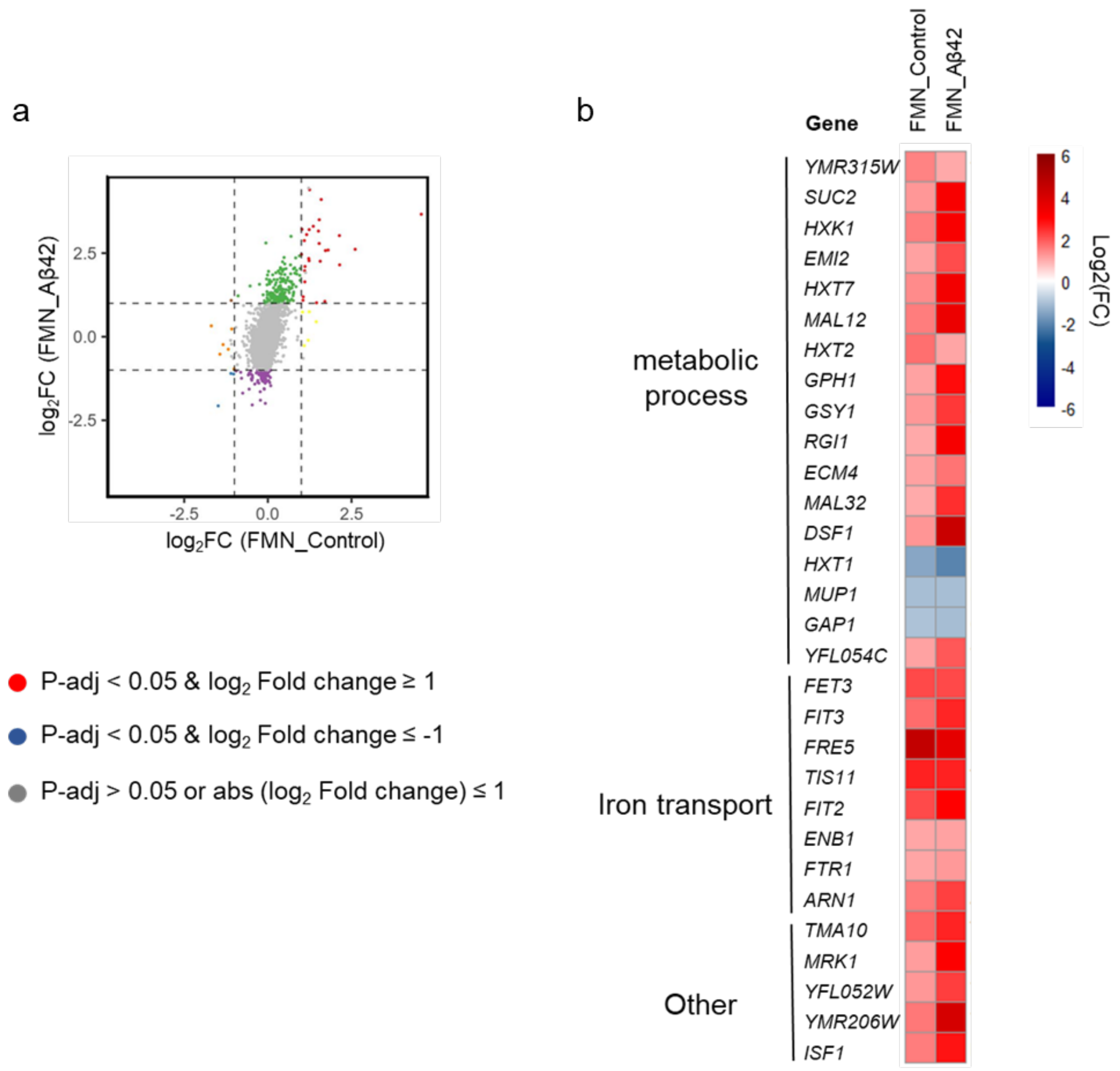

Supplementary Figure 16. Summary of the differentially expressed genes in the control and Aβ42 strains with or without FMN supplementation. (a) X axis represents the $\log_2$ of fold change (FC) in the control strain with FMN supplementation versus without (FMN_control), while the Y axis represents the $\log_2$ of Fold change (FC) in the Aβ42 strain with FMN supplementation versus without (FMN_Aβ42). The highlighted points indicate genes with significantly increased ($\log_2FC \geq 1$, red) or decreased ($\log_2FC \leq -1$, blue) expression, respectively (p-adj < 0.05). (b) The differentially expressed genes (red and blue points from a) are mostly related to metabolic process and iron transport.

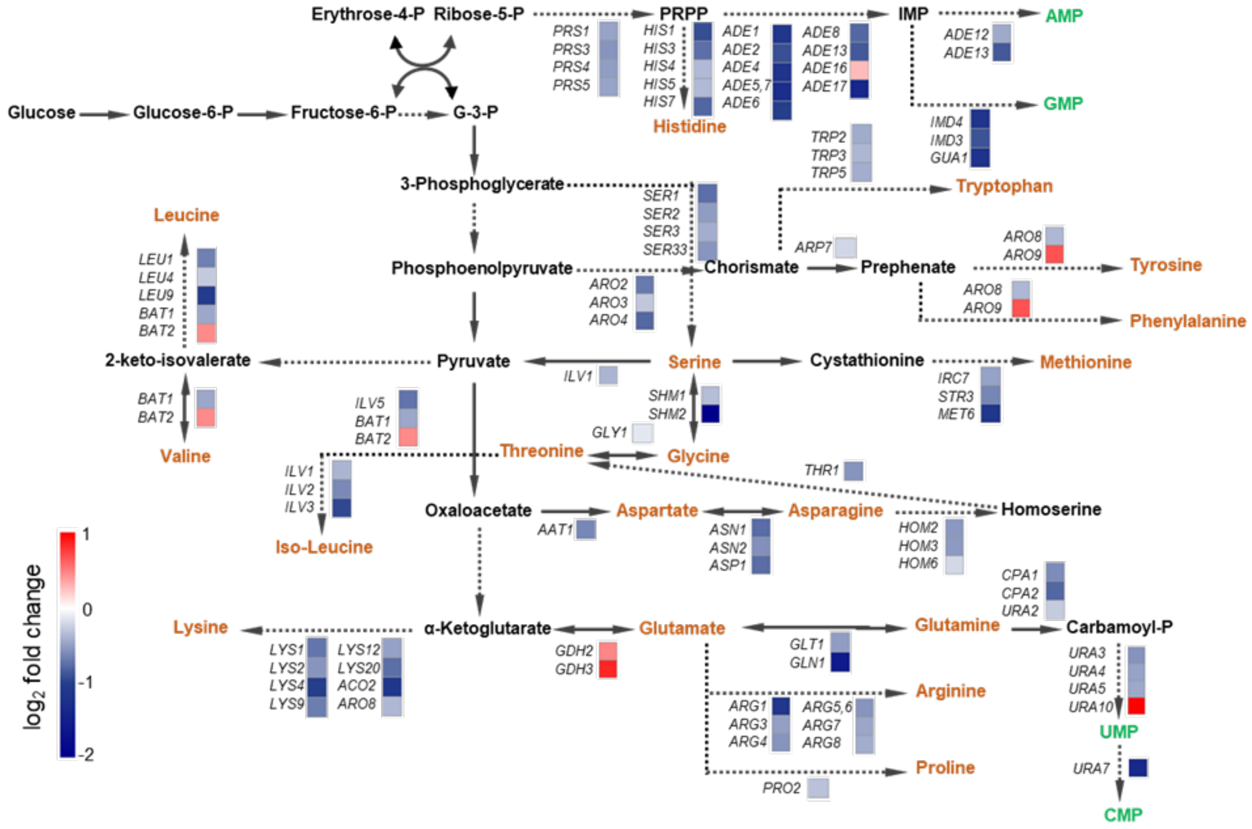

Supplementary Figure 17. Schematic overview of significantly changed genes in amino acid biosynthesis in the Aβ42 strain with or without FMN supplementation (p-adj < 0.05).

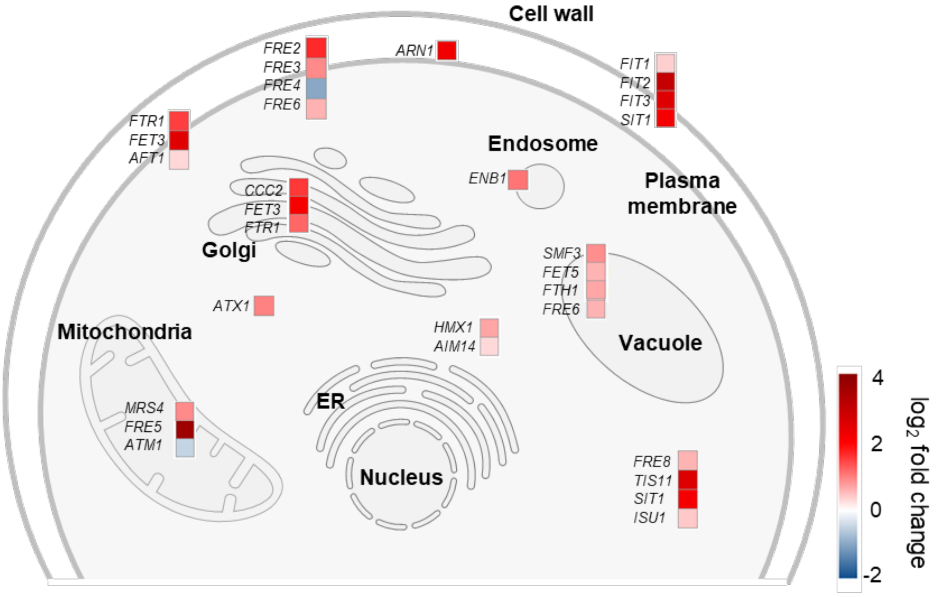

Supplementary Figure 18. Transcriptional changes of genes related to iron transport and iron homeostasis processes in Aβ42 strain with or without FMN supplementation (p-adj < 0.05).

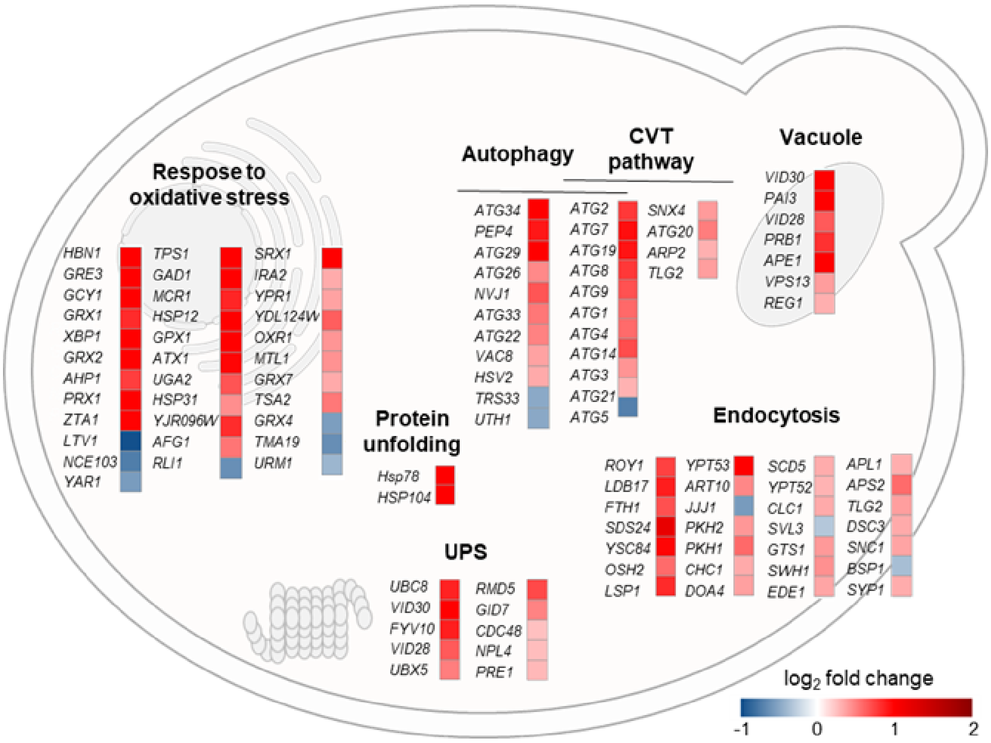

Supplementary Figure 19. Transcriptional changes of genes related to the oxidative stress response and protein metabolic processes in the Aβ42 strain with or without FMN supplementation (p-adj < 0.05).

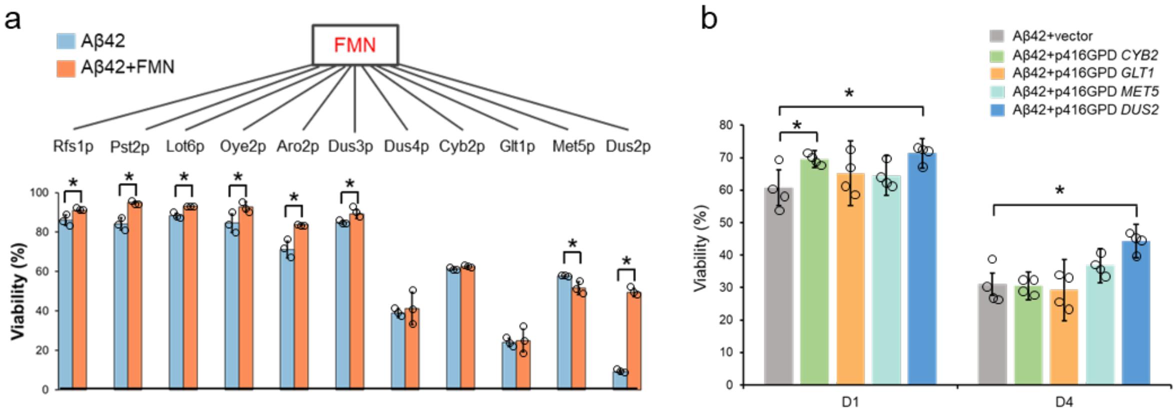

Supplementary Figure 20. Genetic interactions between FMN-dependent flavoproteins and Aβ42. (A) Viability of Aβ42 strains with independent deletions of genes encoding FMN-dependent flavoproteins on day 3. (B) Viability over time of Aβ42 strains with overexpression of genes encoding FMN-dependent flavoproteins on day 1 (D1) and day 4 (D4). Results are average values ± SD of biological triplicates. The asterisk (*) indicates significant differences (p < 0.05). Source data are provided as a Source Data file.

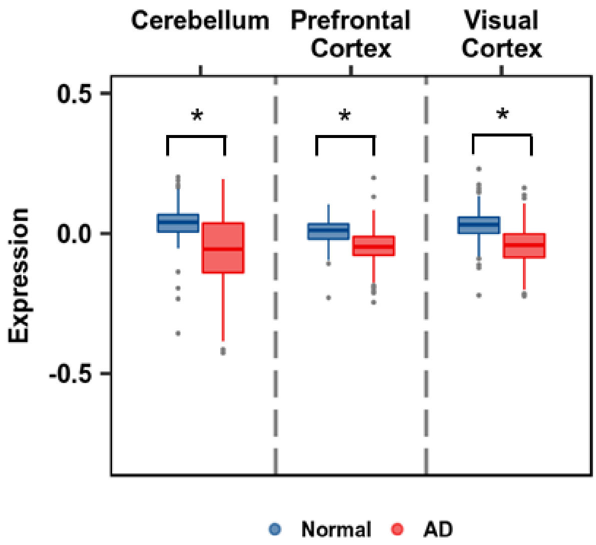

Supplementary Figure 21. The mRNA expression levels of human *DUS2* in the cerebellum, prefrontal cortex and visual cortex of normal controls and AD patients. Asterisks (*) indicate significant differences (p-adj < 0.0001).

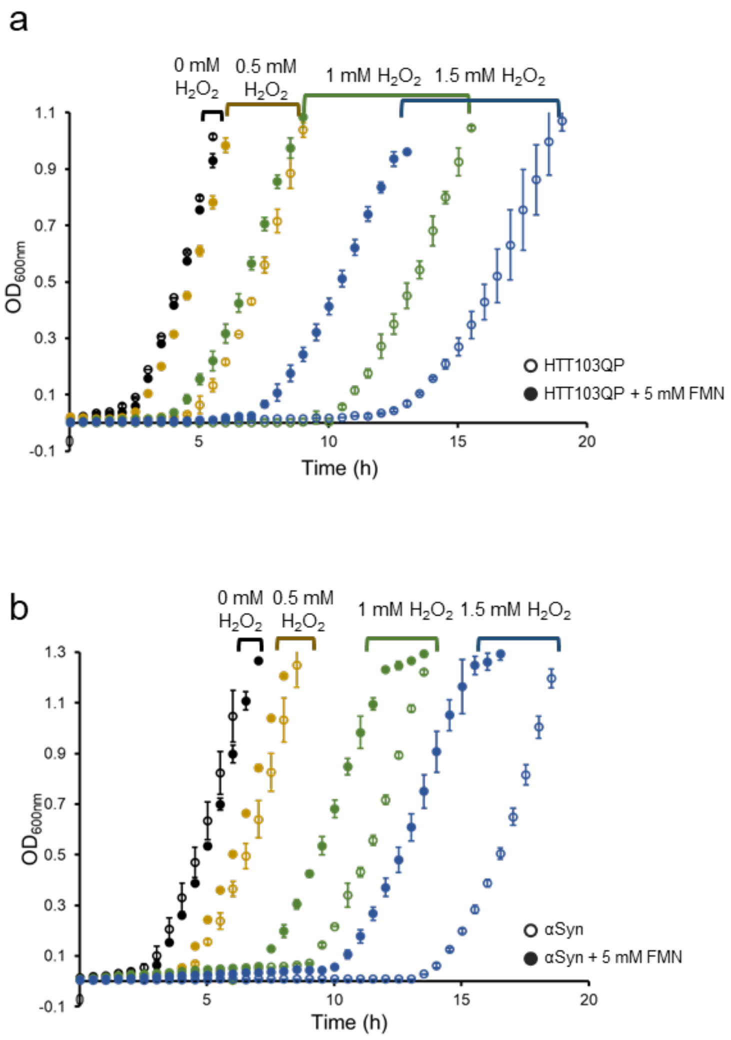

Supplementary Figure 22. HTT103QP strain (a) and the α-synuclein strain (b) display an increased H_2O_2 tolerance with FMN supplementation. Cells without or with 5 mM FMN supplement were grown to OD ≈ 0.2 and treated with different concentrations of H_2O_2. Cell growth was monitored using a microplate reader. Results shown are average values ± SD of biological duplicates. Source data are provided as a Source Data file.

Supplementary Table 1. Distribution of GO terms among the nonessential mutants with significantly altered Aβ42 toxicity via SAFE analysis.

Domain	GO biological process term	Percentage (%)
Sensitivity		
1	other	2.23
2	Glycosylation, Protein Folding/Targeting, Cell Wall Biosynthesis	21.65
3	Ribosome Biogenesis	0.26
4	Protein Degradation	1.71
7	MVB Sorting and pH-dependent Signaling	2.60
12	DNA Replication and Repair	0.26
13	Transcription and Chromatin Organization	0.39
14	Vesicle Traffic	11.81
15	Cell Polarity and Morphogenesis	7.35
16	Mitosis and Chromosome Segregation	1.18
Resistance		
1	other	5.34
2	Glycosylation, Protein Folding/Targeting, Cell Wall Biosynthesis	2.85
3	Ribosome Biogenesis	9.43
4	Protein Degradation	0.71
6	Nuclear-cytoplasmic Transport	1.42
9	tRNA Wobble Modification	0.71
10	Peroxisome	0.53
11	Metabolism and Fatty Acid Biosynthesis	1.78
12	DNA Replication and Repair	0.36
13	Transcription and Chromatin Organization	9.07
15	Cell Polarity and Morphogenesis	7.30
16	Mitosis and Chromosome Segregation	0.71
17	rRNA and ncRNA Processing	1.07
18	Respiration, Oxidative Phosphorylation, Mitochondrial Targeting	8.54

Supplementary Table 2. Distribution of GO terms among the essential mutants with significantly altered Aβ42 toxicity via SAFE analysis.

Domain	GO biological process term	Percentage (%)
Sensitivity		
1	Other	0.93
2	Glycosylation, Protein Folding/Targeting, Cell Wall Biosynthesis	17.06
4	Protein Degradation	5.96
7	MVB Sorting and pH-dependent Signaling	0.35
8	mRNA Processing	0.70
11	Metabolism and Fatty Acid Biosynthesis	0.35
12	DNA Replication and Repair	1.40
13	Transcription and Chromatin Organization	1.17
14	Vesicle Traffic	10.16
15	Cell Polarity and Morphogenesis	10.51
16	Mitosis and Chromosome Segregation	1.17
Resistance		
1	Other	4.00
2	Glycosylation, Protein Folding/Targeting, Cell Wall Biosynthesis	0.72
4	Protein Degradation	0.92
5	Cytokinesis	1.23
6	Nuclear-cytoplasmic Transport	3.39
8	mRNA Processing	0.51
9	tRNA Wobble Modification	0.51
10	Peroxisome	0.62
12	DNA Replication and Repair	5.34
13	Transcription and Chromatin Organization	7.49
14	Vesicle Traffic	0.31
15	Cell Polarity and Morphogenesis	0.92
16	Mitosis and Chromosome Segregation	14.78
17	rRNA and ncRNA Processing	8.21
18	Respiration, Oxidative Phosphorylation, Mitochondrial Targeting	0.62

Supplementary Table 3. List of mutants in the protein secretion and degradation processes that increase Aβ42 toxicity.

Systematic name	Standard name	SGA score of run 1	SGA score of run 2
YHR101C	*BIG1*	-1.377	-1.505
YPR181C	*SEC23*	-1.290	-1.010
YBR160W	*CDC28*	-1.125	-1.002
YMR298W	*LIP1*	-1.065	-0.825
YOR070C	*GYP1*	-1.042	-0.887
YOR336W	*KRE5*	-0.995	-0.957
YGL027C	*CWH41*	-0.965	-0.687
YOL031C	*SIL1*	-0.962	-0.603
YLL031C	*GPI13*	-0.923	-1.002
YML130C	*ERO1*	-0.909	-0.845
YFL031W	*HAC1*	-0.904	-1.005
YBR229C	*ROT2*	-0.895	-1.121
YJL002C	*OST1*	-0.892	-0.916
YOR085W	*OST3*	-0.862	-0.621
YDR182W	*CDC1*	-0.852	-0.821
YNL287W	*SEC21*	-0.831	-0.766
YDL126C	*CDC48*	-0.820	-0.885
YHR030C	*SLT2*	-0.815	-0.766
YJR075W	*HOC1*	-0.811	-0.808
YNL263C	*YIF1*	-0.803	-1.116
YBL040C	*ERD2*	-0.780	-0.974
YDR245W	*MNN10*	-0.779	-1.004
YNL322C	*KRE1*	-0.776	-0.911
YDR414C	*ERD1*	-0.744	-0.585
YNL291C	*MID1*	-0.731	-0.919
YLR262C	*YPT6*	-0.715	-0.915
YMR264W	*CUE1*	-0.712	-0.677
YGR172C	*YIP1*	-0.703	-0.554
YNL307C	*MCK1*	-0.699	-0.679
YGR216C	*GPI1*	-0.687	-0.500
YMR307W	*GAS1*	-0.670	-0.747
YBR036C	*CSG2*	-0.663	-0.711
YER083C	*GET2*	-0.653	-0.763
YJR073C	*OPI3*	-0.635	-0.567
YNL051W	*COG5*	-0.633	-0.546
YJL029C	*VPS53*	-0.624	-0.569
YJR010C-A	*SPC1*	-0.580	-0.788
YCL001W	*RER1*	-0.579	-0.635
YAL053W	*FLC2*	-0.563	-0.504
YGR217W	*CCH1*	-0.557	-0.542
YBR015C	*MNN2*	-0.555	-0.500
YDR236C	*FMN1*	-0.554	-0.656
YAL058W	*CNE1*	-0.545	-0.500
YBR162W-A	*YSY6*	-0.521	-0.685
YDL232W	*OST4*	-0.513	-0.615
YGL055W	*OLE1*	-0.511	-0.639
YEL002C	*WBP1*	-0.500	-0.706

Supplementary Table 4. List of mutants in protein secretion and degradation processes that reduce Aβ42 toxicity.

Systematic name	Standard name	SGA score of run 1	SGA score of run 2
YHR036W	*BRL1*	0.872	0.558
YML105C	*SEC65*	0.852	0.532
YLR100W	*ERG27*	0.707	0.661
YJL085W	*EXO70*	0.705	0.554
YDR437W	*GPI19*	0.642	1.049
YOR254C	*SEC63*	0.623	0.602
YKL154W	*SRP102*	0.541	0.559

Supplementary Table 5. Plasmids used in this study.

Plasmids	Description	Sources
p416 GPD (empty vector)	*CEN*, *Amp*^{R}, *URA3*, *GPD1p*, *CYC1t*	[1]
p416 GPD-Aβ42	*CEN*, *Amp*^{R}, *URA3*, *GPD1p-Aβ42-CYC1t*	[2]
pFA6a-natNT2	*CEN*, *Amp*^{R}, *TEF1p-natNT2-ADH1t*	[3]
pFA6a-hphNT1	*CEN*, *Amp*^{R}, *TEF1p-hphNT1-CYC1t*	[3]
p416 GPD-FMN1	*CEN*, *Amp*^{R}, *URA3*, *GPD1p-FMN1-CYC1t*	This study
p416 GPD-RFK	*CEN*, *Amp*^{R}, *URA3*, *GPD1p-RFK-CYC1t*	This study
p416 GPD-CYB2	*CEN*, *Amp*^{R}, *URA3*, *GPD1p-CYB2-CYC1t*	This study
p416 GPD-GLT1	*CEN*, *Amp*^{R}, *URA3*, *GPD1p-GLT1-CYC1t*	This study
p416 GPD-MET5	*CEN*, *Amp*^{R}, *URA3*, *GPD1p-MET5*-CYC1t	This study
p416 GPD-DUS2	*CEN*, *Amp*^{R}, *URA3*, *GPD1p-DUS2-CYC1t*	This study
pYES2-*HTT103QP*	*2μ*, *Amp*^{R}, *URA3*, *GAL1p-HTT103QP-GFP-CYC1t*	[4]
p416 GPD-*HTT103QP*	*CEN*, *Amp*^{R}, *URA3*, *GPD1p-HTT103QP-GFP-CYC1t*	This study
pUG23 α-syn	*CEN*, *Amp*^{R}, *HIS3*, *MET25p-α-synuclein-EGFP-CYC1t*	[5]
P5586 (MoBY empty vector)	*CEN*, *KanMX*^{R}, *Hygromycin B*^{R}, *Tetracycline*^{R}, *URA3*	[6]
pERD2-MoBY	*CEN*, *KanMX*^{R}, *Hygromycin B*^{R}, *Tetracycline*^{R}, *URA3, ERD2**	[6]
pCDC1-MoBY	*CEN, KanMX*^{R}, *Hygromycin B*^{R}, *Tetracycline*^{R}, *URA3*, *CDC1**	[6]
pFMN1-MoBY	*CEN, KanMX*^{R}, *Hygromycin B*^{R}, *Tetracycline*^{R}, *URA3*, *FMN1**	[6]
pFRQ1-MoBY	*CEN, KanMX*^{R}, *Hygromycin B*^{R}, *Tetracycline*^{R}, *URA3*, *FRQ1**	[6]
pWBP1-MoBY	*CEN, KanMX*^{R}, *Hygromycin B*^{R}, *Tetracycline*^{R}, *URA3*, *WBP1**	[6]
pYIP1-MoBY	*CEN, KanMX*^{R}, *Hygromycin B*^{R}, *Tetracycline*^{R}, *URA3*, *YIP1**	[6]
pGPI1-MoBY	*CEN, KanMX*^{R}, *Hygromycin B*^{R}, *Tetracycline*^{R}, *URA3*, *GPI1**	[6]
pOST1-MoBY	*CEN, KanMX*^{R}, *Hygromycin B*^{R}, *Tetracycline*^{R}, *URA3*, *OST1**	[6]
pGFA1-MoBY	*CEN, KanMX*^{R}, *Hygromycin B*^{R}, *Tetracycline*^{R}, *URA3*, *GFA1**	[6]
pSRP102-MoBY	*CEN, KanMX*^{R}, *Hygromycin B*^{R}, *Tetracycline*^{R}, *URA3*, *SRP102**	[6]
pERG27-MoBY	*CEN, KanMX*^{R}, *Hygromycin B*^{R}, *Tetracycline*^{R}, *URA3*, *ERG27**	[6]
pERO1-MoBY	*CEN, KanMX*^{R}, *Hygromycin B*^{R}, *Tetracycline*^{R}, *URA3*, *ERO1**	[6]
pSEC21-MoBY	*CEN, KanMX*^{R}, *Hygromycin B*^{R}, *Tetracycline*^{R}, *URA3*, *SEC21**	[6]
pKRE5-MoBY	*CEN, KanMX*^{R}, *Hygromycin B*^{R}, *Tetracycline*^{R}, *URA3*, *KRE5**	[6]
pTHI80-MoBY	*CEN, KanMX*^{R}, *Hygromycin B*^{R}, *Tetracycline*^{R}, *URA3*, *THI80**	[6]
pGPI13-MoBY	*CEN, KanMX*^{R}, *Hygromycin B*^{R}, *Tetracycline*^{R}, *URA3*, *GPI13**	[6]
pUPS1-MoBY	*CEN, KanMX*^{R}, *Hygromycin B*^{R}, *Tetracycline*^{R}, *URA3*, *UPS1**	[6]
pOST3-MoBY	*CEN, KanMX*^{R}, *Hygromycin B*^{R}, *Tetracycline*^{R}, *URA3*, *OST3**	[6]
pCNE1-MoBY	*CEN, KanMX*^{R}, *Hygromycin B*^{R}, *Tetracycline*^{R}, *URA3*, *CNE1**	[6]
pCWH41-MoBY	*CEN, KanMX*^{R}, *Hygromycin B*^{R}, *Tetracycline*^{R}, *URA3*, *CWH41**	[6]
pSNF4-MoBY	*CEN, KanMX*^{R}, *Hygromycin B*^{R}, *Tetracycline*^{R}, *URA3*, *SNF4**	[6]
pROT2-MoBY	*CEN, KanMX*^{R}, *Hygromycin B*^{R}, *Tetracycline*^{R}, *URA3*, *ROT2**	[6]
pYDJ1-MoBY	*CEN, KanMX*^{R}, *Hygromycin B*^{R}, *Tetracycline*^{R}, *URA3*, *YDJ1**	[6]
pRER1-MoBY	*CEN, KanMX*^{R}, *Hygromycin B*^{R}, *Tetracycline*^{R}, *URA3*, *RER1**	[6]
pECAS9-gRNA-KanMX-tHFD1	*2μ, Amp*^{R}, *KanMX*^{R}, *TEF1p-eCas9-CYC1t, gRNA-HFD1*	[7]
pECAS9-gRNA-KanMX-XI-3	*2μ, Amp*^{R}, *KanMX*^{R}, *TEF1p-eCas9-CYC1t, gRNA-XI-3*	This study

pECAS9-gRNA-KanMX-XII-5	*2μ*, Amp^R, $KanMX^R$, *TEF1p-eCas9-CYC1t, gRNA-XII-5*	This study

*Gene expression is controlled by its native promoter and terminator.

Supplementary Table 6. Strains used in this study.

Strains	Description	Background	Sources	Experiments and Figures
Y7092	*MATα can1Δ::STE2pr-his5 lyp1Δ ura3Δ0 leu2Δ0 his3Δ1 met15Δ0*	BY4742	[8]	SGA start strain
Control	*MATα can1Δ::STE2pr-his5 lyp1Δ ura3Δ0 leu2Δ0 his3Δ1 met15Δ0 XI-3::GPDp-CYC1t+TEF1p-natNT2-ADH1t XII-5::GPDp-CYC1t+TEF1p-hphNT1-ADH1t*	BY4742	This study	SGA query strain Fig.1, 2a, S1-S6
Aβ42	*MATα can1Δ::STE2pr-his5 lyp1Δ ura3Δ0 leu2Δ0 his3Δ1 met15Δ0 XI-3::GPDp-Aβ42-CYC1t+TEF1p-natNT2-ADH1t XII-5::GPDp-Aβ42-CYC1t+TEF1p-hphNT1-ADH1t*	BY4742	This study	
Aβ42 cwh41Δ	*MATa Aβ42 cwh41Δ::kanMX4*	BY4741	This study	Random spore analysis Table 1
Aβ42 ost3Δ	*MATa Aβ42 ost3Δ::kanMX4*	BY4741	This study	
Aβ42 rer1Δ	*MATa Aβ42 rer1Δ::kanMX4*	BY4741	This study	
Aβ42 cne1Δ	*MATa Aβ42 cne1Δ::kanMX4*	BY4741	This study	
Aβ42 rot2Δ	*MATa Aβ42 rot2Δ::kanMX4*	BY4741	This study	
Aβ42 ydj1Δ	*MATa Aβ42 ydj1Δ::kanMX4*	BY4741	This study	
Aβ42 ups1Δ	*MATa Aβ42 ups1Δ::kanMX4*	BY4741	This study	
Aβ42 snf4Δ	*MATa Aβ42 snf4Δ::kanMX4*	BY4741	This study	
Aβ42 ero1	*MATa Aβ42 ero1::kanMX4*	BY4741	This study	
Aβ42 ost1	*MATa Aβ42 ost1::kanMX4*	BY4741	This study	
Aβ42 erd2	*MATa Aβ42 erd2::kanMX4*	BY4741	This study	
Aβ42 cdc1	*MATa Aβ42 cdc1::kanMX4*	BY4741	This study	
Aβ42 kre5	*MATa Aβ42 kre5::kanMX4*	BY4741	This study	
Aβ42 sec21	*MATa Aβ42 sec21::kanMX4*	BY4741	This study	
Aβ42 fmn1	*MATa Aβ42 fmn1::kanMX4*	BY4741	This study	
Aβ42 gpi13	*MATa Aβ42 gpi13::kanMX4*	BY4741	This study	
Aβ42 gpi1	*MATa Aβ42 gpi1::kanMX4*	BY4741	This study	
Aβ42 yip1	*MATa Aβ42 yip1::kanMX4*	BY4741	This study	
Aβ42 erg27	*MATa Aβ42 erg27::kanMX4*	BY4741	This study	
Aβ42 frq1	*MATa Aβ42 frq1::kanMX4*	BY4741	This study	
Aβ42 srp102	*MATa Aβ42 srp102::kanMX4*	BY4741	This study	
Aβ42 gfa1	*MATa Aβ42 gfa1::kanMX4*	BY4741	This study	
Aβ42 thi80	*MATa Aβ42 thi80::kanMX4*	BY4741	This study	
Aβ42 cwh41Δ CWH41	*MATa Aβ42 cwh41Δ::kanMX4 pCWH41-MoBY*	BY4741	This study	
Aβ42 ost3Δ OST3	*MATa Aβ42 ost3Δ::kanMX4 pOST3-MoBY*	BY4741	This study	Complement-ation assay Fig. 3b,3d, S8, S9
Aβ42 rer1Δ RER1	*MATa Aβ42 rer1Δ::kanMX4 pRER1-MoBY*	BY4741	This study	
Aβ42 cne1Δ CNE1	*MATa Aβ42 cne1Δ::kanMX4 pCNE1-MoBY*	BY4741	This study	
Aβ42 rot2Δ ROT2	*MATa Aβ42 rot2Δ::kanMX4 pROT2-MoBY*	BY4741	This study	
Aβ42 ydj1Δ YDJ1	*MATa Aβ42 ydj1Δ::kanMX4 pYDJ1-MoBY*	BY4741	This study	
Aβ42 ups1Δ UPS1	*MATa Aβ42 ups1Δ::kanMX4 pUPS1-MoBY*	BY4741	This study	
Aβ42 snf4Δ SNF4	*MATa Aβ42 snf4Δ::kanMX4 pSNF4-MoBY*	BY4741	This study	
Aβ42 ero1 ERO1	*MATa Aβ42 ero1::kanMX4 pERO1-MoBY*	BY4741	This study	
Aβ42 ost1 OST1	*MATa Aβ42 ost1::kanMX4 pOST1-MoBY*	BY4741	This study	
Aβ42 erd2 ERD2	*MATa Aβ42 erd2::kanMX4 pERD2-MoBY*	BY4741	This study	
Aβ42 cdc1 CDC1	*MATa Aβ42 cdc1::kanMX4 pCDC1-MoBY*	BY4741	This study	

Aβ42 kre5 KRE5	*MATa Aβ42 kre5::kanMX4 pKRE5-MoBY*	BY4741	This study	
Aβ42 sec21 SEC21	*MATa Aβ42 sec21::kanMX4 pSEC21-MoBY*	BY4741	This study	
Aβ42 fmn1 FMN1	*MATa Aβ42 fmn1::kanMX4 pFMN1-MoBY*	BY4741	This study	
Aβ42 gpi13 GPI13	*MATa Aβ42 gpi13::kanMX4 pGPI13-MoBY*	BY4741	This study	
Aβ42 gpi1 GPI1	*MATa Aβ42 gpi1::kanMX4 pGPI1-MoBY*	BY4741	This study	
Aβ42 yip1 YIP1	*MATa Aβ42 yip1::kanMX4 pYIP1-MoBY*	BY4741	This study	
Aβ42 erg27 ERG27	*MATa Aβ42 erg27::kanMX4 pERG27-MoBY*	BY4741	This study	
Aβ42 frq1 FRQ1	*MATa Aβ42 frq1::kanMX4 pFRQ1-MoBY*	BY4741	This study	
Aβ42 srp102 SRP102	*MATa Aβ42 srp102::kanMX4 pSRP102-MoBY*	BY4741	This study	
Aβ42 gfa1 GFA1	*MATa Aβ42 gfa1::kanMX4 pGFA1-MoBY*	BY4741	This study	
Aβ42 thi80 THI80	*MATa Aβ42 thi80::kanMX4 pTHI80-MoBY*	BY4741	This study	
WT Hsp104-GFP	*MATa his3Δ::kanMX4 can1Δ::STE2pr-Sp_his5 lyp1Δ ura3Δ0 met15Δ0 HSP104-GFP-LEU2*	BY4741	SGA library[9]	Inclusion body morphology test
fmn1	*fmn1::KanMX4*	BY4741	TS-V6 collection	Fig. 4a
fmn1 Hsp104-GFP	*MATa his3Δ0 fmn1::kanMX4 can1Δ::STE2pr-Sp_his5 lyp1Δ ura3Δ0 met15Δ0 HSP104-GFP-LEU2*	BY4741	This study	
Control (CEN)	*MATa his3Δ1 ura3-52 MAL2-8c SUC2 XI-3::GPDp-CYC1t XII-5::GPDp-CYC1t*	CEN.PK 113-11C	This study	Growth rate, NADP(H) and
Aβ42 (CEN)	*MATa his3Δ1 ura3-52 MAL2-8c SUC2 XI-3::GPDp-Aβ42-CYC1t XII-5::GPDp-Aβ42-CYC1t*	CEN.PK 113-11C	This study	NAD(H) measurement, H_2O_2 treatment, RNA-seq, qPCR, viability, AZE treatment, western blot Fig.3c, 3e, 3f, 4b,4c,4d, 5, 6, S10-S19
rfs1Δ	*rfs1::KanMX4*	BY4741	SGA-V2	Flavoprotein
pst1Δ	*pst2::KanMX4* Aβ42 (CEN)	BY4741	SGA-V2	experiments
cyb2Δ	*cyb2::KanMX4*	BY4741	SGA-V2	Fig. S20
lot6Δ	*lot6Δ::KanMX4*	BY4741	SGA-V2	
dus2Δ	*dus2Δ::KanMX4*	BY4741	SGA-V2	
oye2Δ	*oye2Δ::KanMX4*	BY4741	SGA-V2	
aro2Δ	*aro2Δ::KanMX4*	BY4741	SGA-V2	
glt1Δ	*glt1Δ::KanMX4*	BY4741	SGA-V2	
dus3Δ	*dus3Δ::KanMX4*	BY4741	SGA-V2	
dus4Δ	*dus4Δ::KanMX4*	BY4741	SGA-V2	
met5Δ	*met5Δ::KanMX4*	BY4741	SGA-V2	
Aβ42 rfs1Δ	*rfs1Δ::kanMX4 p416 GPD-Aβ42*	BY4741	This study	
Aβ42 pst1Δ	*pst1Δ::kanMX4 p416 GPD-Aβ42*	BY4741	This study	
Aβ42 cyb2Δ	*cyb2Δ::kanMX4 p416 GPD-Aβ42*	BY4741	This study	
Aβ42 lot6Δ	*lot6Δ::kanMX4 p416 GPD-Aβ42*	BY4741	This study	
Aβ42 dus2Δ	*dus2Δ::kanMX4 p416 GPD-Aβ42*	BY4741	This study	
Aβ42 oye2Δ	*oye2Δ::kanMX4 p416 GPD-Aβ42*	BY4741	This study	

Aβ42 aro2Δ	*aro2Δ::kanMX4 p416 GPD-Aβ42*	BY4741	This study	
Aβ42 glt1Δ	*glt1Δ::kanMX4 p416 GPD-Aβ42*	BY4741	This study	
Aβ42 dus3Δ	*dus3Δ::kanMX4 p416 GPD-Aβ42*	BY4741	This study	
Aβ42 dus4Δ	*dus4Δ::kanMX4 p416 GPD-Aβ42*	BY4741	This study	
Aβ42 met5Δ	*met5Δ::kanMX4 p416 GPD-Aβ42*	BY4741	This study	
Aβ42 DUS2	*Aβ42 (CEN) p416 GPD-DUS2*	CEN.PK 113-11C	This study	
Aβ42 CYB2	*Aβ42 (CEN) p416 GPD-CYB2*	CEN.PK 113-11C	This study	
Aβ42 MET5	*Aβ42 (CEN) p416 GPD-MET5*	CEN.PK 113-11C	This study	
Aβ42 GLT1	*Aβ42 (CEN) p416 GPD-GLT1*	CEN.PK 113-11C	This study	
*HTT*103QP	*MATa his3Δ1 ura3-52 MAL2-8c SUC2 p416 GPD-HTT103QP*	CEN.PK 113-11C	This study	H_2O_2 treatment, viability
α-syn	*MATa his3Δ1 ura3-52 MAL2-8c SUC2 pUG23 α-syn*	CEN.PK 113-11C	This study	Fig. 7, S22

Supplementary Table 7. Primers used in this study.

Primers	Sequence (5’-3’)
XI-3-up-L1	AGTTACTTGCTCTATGCGTTTGCGC
XI-3-up-R1	GTATTCTTTGAAATGGCGAGTATTGATAATGATAAACTAATCAGACGCACGCTTGGCGTC
Aβ42-L	AGTTTATCATTATCAATACTCGCCATTTC
Aβ42-R	GCAAATTAAAGCCTTCGAGCGTCCC
NatNT2-L	GAGAAGGTTTTGGGACGCTCGAAGGCTTTAATTTGCCGTACGCTGCAGGTCGACGGATC
NatNT2-R	GTTTAATAATGATCTGTATTGCTGGCTCAATCCACGTAAATCGATGAATTCGAGCTCG
XI-3-down-L	TTACGTGGATTGAGCCAGCAATAC
XI-3-down-R	GAGAATCCGGACCAGCAGATAATG
XII-5-up-L	CAATCTGGCGGCTTGAGTTCTCAAC
XII-5-up-R	GAGAAGGTTTTGGGACGCTCGAAGGCTTTAATTTGCTCAAACCGGTTATAGCGGTCTCC
HphNT1-L	CTTTGAAATGGCGAGTATTGATAATGATAAACTCGTACGCTGCAGGTCGACGG
HphNT1-R	CTTATTTAATAATAAAAATCATAAATCATAAGAAATTCGCTTATTCCTTTGCCCTCGGACGAG
ADHt-L	GCGAATTTCTTATGATTTATGATTTTT
ADHt-R	GCATATCTACAATTGGGTGAAATGG
XII-5-down-L1	CTGCAAATCGCTCCCCATTTCACCCAATTGTAGATATGCGCAACTCAGAAGTTTGACAGCAAGC
XII-5-down-R	CTGCGATACCTTTTGTGATGGCTAAAC
XII-5-down-L2	CTTTGAAATGGCGAGTATTGATAATGATAAACTGCAACTCAGAAGTTTGACAGCAAGC
XI-3-down-L2	GAGAAGGTTTTGGGACGCTCGAAGGCTTTAATTTGCTTACGTGGATTGAGCCAGCAATACAG
XI-3-down-R2	GAGAATCCGGACCAGCAGATAATGCATGC
FMN1-L1	CGCGAATTCAACAAAATGTTTACGTGGACTATTTATGTTTCG
FMN1-R1	CCCAAGCTTCTATAATTGTTGTTTGAATACTTG
ACT-L	GCCTTCTACGTTTCCATCCA
ACT-R	GGCCAAATCGATTCTCAAAA
FMN1-L2	TCGATTTGCCAATACCTGCG
FMN1-R2	CGCCTTGTTTCCACCGATAG
RFK-L	CGCGAATTCAACAAAATGAGACATTTGCCTTATTTTTG
RFK-R	CCCAAGCTTTTAGTGACCGTTCATTATTTTGG
CAS9-L1	GAACGGTCGAGAAGCTCATA
CAS9-R1	TAAGTTGGTACCCGAGGTAC
CAS9-L2	TGCGGCGTACATCACTTTGT
CAS9-R2	TATGAGCTTCTCGACCGTTC
P297	ATATGTCTCTAATTTTGGAAGTTTTAGAGCTAGAAATAGC
P298	TTCCAAAATTAGAGACATATGATCATTTATCTTTCACTGC
P299	TTGTCACAGTGTCACATCAGGTTTTAGAGCTAGAAATAGC
P300	CTGATGTGACACTGTGACAAGATCATTTATCTTTCACTGC
Hsp104-GFP-L	CAAGAATTTGGTCATGGGTGCTG
Hsp104-GFP-R	CGTTGGAAATCATCGCTGTTCG
pBACKbone-F	TGATAATCTCATGACCAAAATCCC
pBACKbone-R	GAACGAAAACTCACGTTAAGGG
tCYC1-F	CTCGAGTCATGTAATTAGTTATGTC
GAP-R	TCGAAACTAAGTTCTGGTGTTTTAA
GAP-dus2-F	CTTTTTTTTAGTTTTAAAACACCAGAACTTAGTTTCGAATGGTTACATATGCTGGAAAAC
tCYC1-dus2-R	GAATGTAAGCGTGACATAACTAATTACATGACTCGAGTTATATATCTGTGGGAAGGGGTAC
GAP-cyb2-F	CTTTTTTTTAGTTTTAAAACACCAGAACTTAGTTTCGAATGCTAAAATACAAACCTTTACTAAA
tCYC1-cyb2-R	ATGTAAGCGTGACATAACTAATTACATGACTCGAGTCATGCATCCTCAAATTCTGTTAA
GAP-glt1-F	CTTTTTTTTAGTTTTAAAACACCAGAACTTAGTTTCGAATGCCAGTGTTGAAATCAGACA
tCYC1-glt1-R	GAATGTAAGCGTGACATAACTAATTACATGACTCGAGTTAGACTTGACTAGCTAATTCTTTCA
GAP-met5-F	CTTTTTTTTAGTTTTAAAACACCAGAACTTAGTTTCGAATGACTGCTTCTGACCTCTTG
tCYC1-met5-R	GAATGTAAGCGTGACATAACTAATTACATGACTCGAGTTAATAGGCATCTTCAGACACAT
GAP-	CTTTTTTTTAGTTTTAAAACACCAGAACTTAGTTTCGAATGGACTACAAGGACGACGAT

HTT103Q-F	
tCYC1-HTT103Q-R	GAATGTAAGCGTGACATAACTAATTACATGACTCGAGTTACTTGTACAGCTCGTCCAT

Supplementary References

1. Mumberg D, Muller R, Funk M. Yeast vectors for the controlled expression of heterologous proteins in different genetic backgrounds. *Gene* **156**, 119-122 (1995).

2. Chen X, Petranovic D. Amyloid-beta peptide-induced cytotoxicity and mitochondrial dysfunction in yeast. *FEMS yeast research* **15**, (2015).

3. Janke C*, et al.* A versatile toolbox for PCR-based tagging of yeast genes: new fluorescent proteins, more markers and promoter substitution cassettes. *Yeast (Chichester, England)* **21**, 947-962 (2004).

4. Berglund LL, Hao X, Liu B, Grantham J, Nyström T. Differential effects of soluble and aggregating polyQ proteins on cytotoxicity and type-1 myosin-dependent endocytosis in yeast. *Scientific Reports* **7**, 11328 (2017).

5. Zabrocki P*, et al.* Characterization of α-synuclein aggregation and synergistic toxicity with protein tau in yeast. **272**, 1386-1400 (2005).

6. Ho CH*, et al.* A molecular barcoded yeast ORF library enables mode-of-action analysis of bioactive compounds. *Nature biotechnology* **27**, 369-377 (2009).

7. Zhu Z, Zhou YJ, Kang M-K, Krivoruchko A, Buijs NA, Nielsen J. Enabling the synthesis of medium chain alkanes and 1-alkenes in yeast. *Metabolic Engineering* **44**, 81-88 (2017).

8. Tong AHY*, et al.* Systematic Genetic Analysis with Ordered Arrays of Yeast Deletion Mutants. *Science* **294**, 2364 (2001).

9. Hill Sandra M*, et al.* Asymmetric Inheritance of Aggregated Proteins and Age Reset in Yeast Are Regulated by Vac17-Dependent Vacuolar Functions. *Cell Reports* **16**, 826-838 (2016).
