# Supplementary material for: FMN reduces Amyloid-β toxicity in yeast by regulating redox status and cellular metabolism
Source: Nat Commun. 2020 Feb 13;11:867. doi: 10.1038/s41467-020-14525-4 (PMC7018843; doi:10.1038/s41467-020-14525-4)
Supplement: Supplementary file 2 — Description of Additional Supplementary Files [file 41467_2020_14525_MOESM2_ESM.docx]

**Description of Supplementary Files**

**File Name: Supplementary Data 1**

**Description:** A list of the positive and negative interactions derived from the nonessential array screen in this study (p-adj < 0.05, scored ≥ 0.2 or ≤ -0.2).

**File Name: Supplementary Data 2**

**Description:** A list of the positive and negative interactions derived from the essential array screen in this study (p-adj < 0.05, scored ≥ 0.2 or ≤ -0.2).

**File Name: Supplementary Data 3**

**Description:** Genes with significantly differential expression in Aβ42 strain upon FMN supplementation from RNA-seq data in this study (p-adj < 0.05).

**File Name: Supplementary Data 4**

**Description:** Genes with significantly differential expression in control strain upon FMN supplementation from RNA-seq data in this study (p-adj < 0.05).
