# Supplementary material for: FMN reduces Amyloid-β toxicity in yeast by regulating redox status and cellular metabolism
Source: Nat Commun. 2020 Feb 13;11:867. doi: 10.1038/s41467-020-14525-4 (PMC7018843; doi:10.1038/s41467-020-14525-4)
Supplement: Supplementary file 7 — Reporting Summary [file 41467_2020_14525_MOESM7_ESM.pdf]

nature research

Corresponding author(s): Dina Petranovic

Last updated by author(s): Dec 18, 2019

Reporting Summary

Nature Research wishes to improve the reproducibility of the work that we publish. This form provides structure for consistency and transparency in reporting. For further information on Nature Research policies, see Authors & Referees and the Editorial Policy Checklist .

Statistics

For all statistical analyses, confirm that the following items are present in the figure legend, table legend, main text, or Methods section.

n/a	Confirmed	
☐	☒	The exact sample size (*n*) for each experimental group/condition, given as a discrete number and unit of measurement
☐	☒	A statement on whether measurements were taken from distinct samples or whether the same sample was measured repeatedly
☐	☒	The statistical test(s) used AND whether they are one- or two-sided<br>*Only common tests should be described solely by name; describe more complex techniques in the Methods section.*
☒	☐	A description of all covariates tested
☐	☒	A description of any assumptions or corrections, such as tests of normality and adjustment for multiple comparisons
☐	☒	A full description of the statistical parameters including central tendency (e.g. means) or other basic estimates (e.g. regression coefficient) AND variation (e.g. standard deviation) or associated estimates of uncertainty (e.g. confidence intervals)
☐	☒	For null hypothesis testing, the test statistic (e.g. *F*, *t*, *r*) with confidence intervals, effect sizes, degrees of freedom and *P* value noted<br>*Give P values as exact values whenever suitable.*
☒	☐	For Bayesian analysis, information on the choice of priors and Markov chain Monte Carlo settings
☒	☐	For hierarchical and complex designs, identification of the appropriate level for tests and full reporting of outcomes
☒	☐	Estimates of effect sizes (e.g. Cohen's *d*, Pearson's *r*), indicating how they were calculated

Our web collection on statistics for biologists contains articles on many of the points above.

Software and code

Policy information about availability of computer code

Data collection	This study included brain mRNA expression datasets information from the GeneNetwork (http://www.genenetwork.org). The microarray data and RNA-seq data from this study can be downloaded from the Genome Expression Omnibus website (GEO, http://www.ncbi.nlm.nih.gov/geo/) with series number GSE94793 and GSE128905, individually.
Data analysis	We applied the data analysis through Nfcore-RNA-seq pipeline (http://github.com/nf-core/rnaseq), Platform for Integrative Analysis of Omics (PIANO) R package, Database for Annotation, Visualization and Integrated Discovery (DAVID, https://david.ncifcrf.gov/), pheatmap R package, R/growthrates package, Spatial Analysis of Functional Enrichment (SAFE) package. The differential gene expression and significance were calculated by the Benjamini-Hochberg method.

For manuscripts utilizing custom algorithms or software that are central to the research but not yet described in published literature, software must be made available to editors/reviewers. We strongly encourage code deposition in a community repository (e.g. GitHub). See the Nature Research guidelines for submitting code & software for further information.

Data

Policy information about availability of data

All manuscripts must include a data availability statement. This statement should provide the following information, where applicable:

- Accession codes, unique identifiers, or web links for publicly available datasets
- A list of figures that have associated raw data
- A description of any restrictions on data availability

The raw RNA-seq data from this study are available at the Genome Expression Omnibus website (GEO, http://www.ncbi.nlm.nih.gov/geo/) with series number GSE128905.

Field-specific reporting

Please select the one below that is the best fit for your research. If you are not sure, read the appropriate sections before making your selection.

☒ Life sciences ☐ Behavioural & social sciences ☐ Ecological, evolutionary & environmental sciences

For a reference copy of the document with all sections, see nature.com/documents/nr-reporting-summary-flat.pdf

Life sciences study design

All studies must disclose on these points even when the disclosure is negative.

Sample size	A minimum of 500 cells were examined for each sample for fluorescent microscopy analyses, which was considered as sufficient to represent the sample with 1x10^9 cells (confidence level 95%, confidence interval 5%).
Data exclusions	No data were excluded from our analyses, except for RNAseq data processing, in which a quality check was performed using FastQC V0.11.7.
Replication	The experiments were performed in three biological replicates unless specified explicity. Significance of differences between strains were determined as mean ± SD using two-tailed student t-test.
Randomization	Our samples were not allocated into experimental groups.
Blinding	The blinding is not relevant for our study, our study was performed in yeast Saccharomyces cerevisiae.

Reporting for specific materials, systems and methods

We require information from authors about some types of materials, experimental systems and methods used in many studies. Here, indicate whether each material, system or method listed is relevant to your study. If you are not sure if a list item applies to your research, read the appropriate section before selecting a response.

Materials & experimental systems

n/a	Involved in the study	
☐	☒	Antibodies
☐	☒	Eukaryotic cell lines
☒	☐	Palaeontology
☒	☐	Animals and other organisms
☒	☐	Human research participants
☒	☐	Clinical data

Methods

n/a	Involved in the study	
☒	☐	ChIP-seq
☐	☒	Flow cytometry
☒	☐	MRI-based neuroimaging

Antibodies

Antibodies used	We used two primary antibodies: mouse monoclonal anti-Aβ antibody (6E10, SIG-39320, Covance) and mouse monoclonal anti-GAPDH antibody (sc-365062, Santa Cruz Biotechnology). The secondary antibody is anti-mouse immunoglobulins-HRP (P0447, Dako).
Validation	The antibody dilution 1:2000 was used for both primary antibodies.

Eukaryotic cell lines

Policy information about cell lines

Cell line source(s)	Yeast Saccharomyces cerevisiae
Authentication	BY4741, BY4742, CEN.PK.113.11C
Mycoplasma contamination	No contamination
Commonly misidentified lines (See ICLAC register)	*Name any commonly misidentified cell lines used in the study and provide a rationale for their use.*

Flow Cytometry

Plots

Confirm that:

- [x] The axis labels state the marker and fluorochrome used (e.g. CD4-FITC).
- [x] The axis scales are clearly visible. Include numbers along axes only for bottom left plot of group (a 'group' is an analysis of identical markers).
- [ ] All plots are contour plots with outliers or pseudocolor plots.
- [x] A numerical value for number of cells or percentage (with statistics) is provided.

Methodology

Sample preparation	Experiments were performed in biological triplicates. Viability was measured by propidium iodine (PI, Thermo Fisher Scientific) staining. 0.5 OD600nm of cells were stained with 0.5 µg/ml of PI in the dark at room temperature for 20 min.
Instrument	Guava easyCyte HT system
Software	FlowJo
Cell population abundance	5000 cells were analyzed for each sample.
Gating strategy	PI is a vital stain which stains dead cells in red color. Two populations could be distinguished based on RFP fluorescence intensity. Positively stained cells fall within the more fluorescent population (mean fluorescence 5–100 times higher).

- [x] Tick this box to confirm that a figure exemplifying the gating strategy is provided in the Supplementary Information.
